# Norepinephrine and dopamine sensor crosstalk depends on local innervation density

**DOI:** 10.1038/s41593-026-02379-w

**Published:** 2026-08-05

**Authors:** Ricardo C. López, Natalie Noble, Özge D. Özçete, Leonardo Silenzi, Xintong Cai, Gillian E. Handy, Jonathan W. Andersen, Tommaso Patriarchi, Yulong Li, Pascal S. Kaeser

**Affiliations:** 1 Department of Neurobiology, Harvard Medical School, Boston, MA USA; 2 Institute of Pharmacology and Toxicology, University of Zürich, Zürich, Switzerland; 3 Neuroscience Center Zürich, ETH and University of Zürich, Zürich, Switzerland; 4 State Key Laboratory of Membrane Biology, Peking University School of Life Sciences, Beijing, China

**Keywords:** Cellular neuroscience, Neurotransmitters, Motor control, Imaging

## Abstract

G-protein-coupled receptor-based fluorescent sensors are widely used to correlate neuromodulatory signaling with brain function. Although experiments in transfected cells often reveal selectivity for individual neurotransmitters, sensor specificity in the brain frequently remains uncertain. Using imaging in mouse brains, we find that norepinephrine and dopamine cross-activate the respective sensors. Non-specific activation occurred when innervation of the cross-reacting transmitter was high, and silencing of specific innervation was indispensable for interpreting sensor fluorescence.

## Abstract (web summary)

This study shows that GPCR-based norepinephrine and dopamine sensors display crosstalk in several brain areas. Relative innervation densities determine the extent of crosstalk, and loss-of-function controls are required to interpret sensor signals.

## Main

G-protein-coupled receptor (GPCR)-based fluorescent sensors have revolutionized the study of neuromodulatory signaling. Their ability to discriminate neurotransmitters relies on the properties of endogenous GPCRs used for sensor engineering. Studies describing new sensors typically measure half-maximal effective concentration (EC_50_) values in transfected cells by puffing neurotransmitters at increasing concentrations^1–6^. Although this approach generally finds high specificity, it is often unclear how this in vitro selectivity translates to the brain. Local innervation densities of neuromodulatory axons and the organization of release and reuptake machinery may strongly influence which transmitters an indicator reports, especially for structurally similar transmitters.

**Fig. 1:**
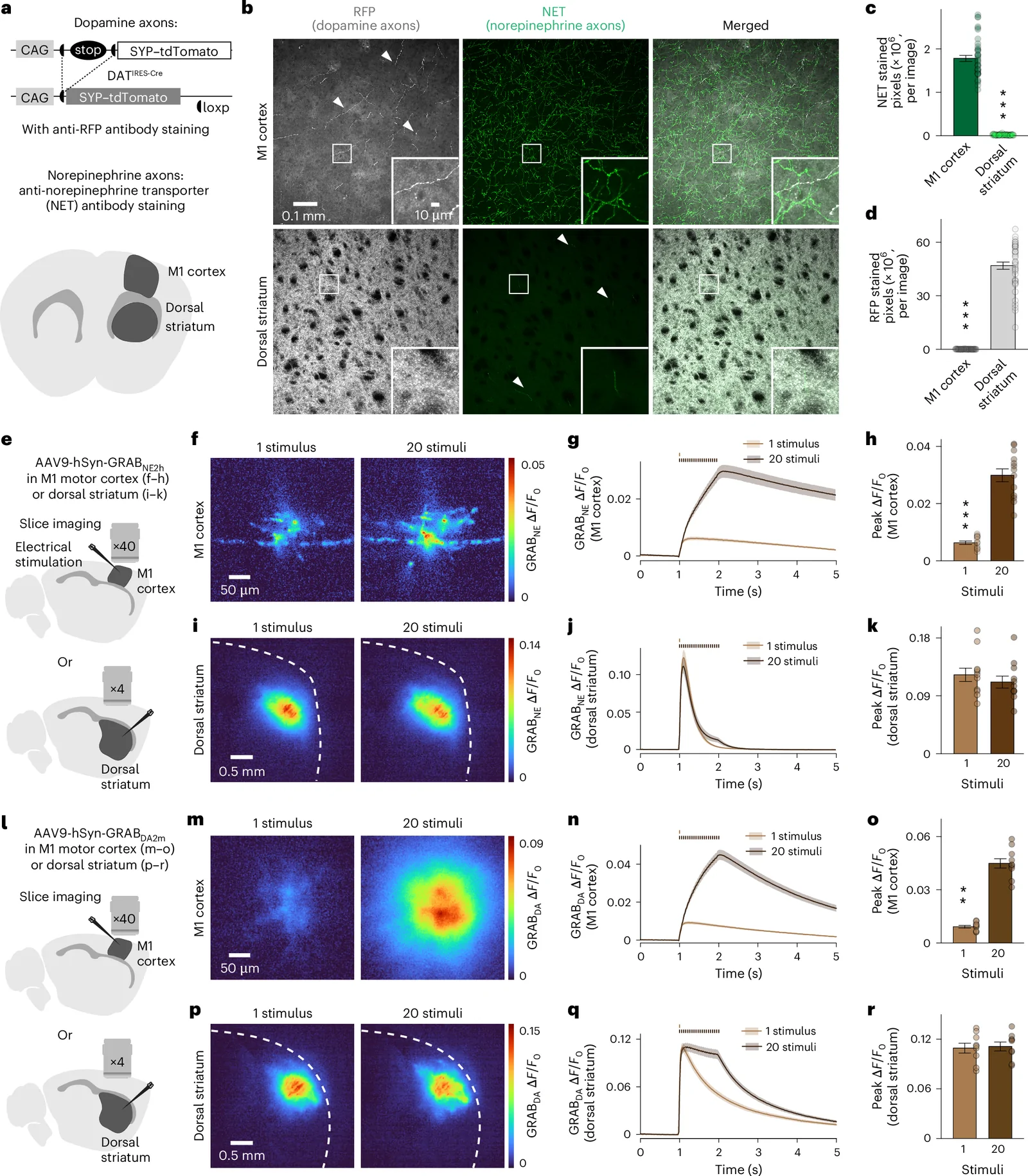
Distinct dopamine and norepinephrine innervation and GRAB_NE_ and GRAB_DA_ dynamics in cortex and striatum. **a**, Strategy for quantification of dopamine and norepinephrine innervation with confocal microscopy. Dopamine axons were labeled by anti-red fluorescent protein (RFP) antibody staining in coronal brain slices of DAT^IRES-Cre^ mice with Cre-dependent synaptophysin–tdTomato (SYP–tdTomato) expression. Norepinephrine axons were labeled with anti-NET antibodies. **b**–**d**, Representative images (**b**) and quantification of NET (**c**) and RFP (**d**) staining in M1 cortex and dorsal striatum. In **b**, brightness and contrast in the gray (RFP) channel were adjusted to render cortical dopamine axons visible and to prevent saturation of dorsal striatum. Arrowheads indicate RFP (M1 cortex) or NET (dorsal striatum) staining. In **c** and **d**, quantification shows the total number of NET-stained or RFP-stained positive pixels in image stacks after binarization: M1 cortex, 40 slices from eight mice; dorsal striatum, 40 slices from eight mice. **e**, Schematic of imaging in acute sagittal slices containing M1 cortex and dorsal striatum. Wide-field fluorescence imaging was performed 3 weeks after stereotaxic AAV injections in the corresponding brain areas to express GRAB_NE_. Electrical stimulation was used to elicit fluorescence transients. **f**–**k**, Representative images of peak GRAB_NE_ fluorescence in response to one stimulus or 20 stimuli at 20 Hz in M1 cortex (**f**) or dorsal striatum (**i**) and quantification of GRAB_NE_ ∆*F*/*F*_0_ time series (**g**,**j**) and peak ∆*F*/*F*_0_ (**h**,**k**): M1 cortex, 13 slices from three mice; dorsal striatum, 11 slices from three mice. **l**–**r**, As in **e**–**k**, but with GRAB_DA_ instead of GRAB_NE_: M1 cortex, ten slices from three mice; dorsal striatum, nine slices from three mice. Data are mean ± s.e.m.; ***P < 0.001; **P < 0.01, as assessed by two-tailed Mann–Whitney rank-sum test in **c** and **d**, and two-tailed Wilcoxon signed-rank test in **h** and **o**. For assessment of somatic stainings in LC and substantia nigra pars compacta, and for the workflow for innervation density analyses, see Extended Data Fig. 1. For antibody controls, see Extended Data Fig. 2. For pharmacological experiments in brain slices, see Extended Data Fig. 3. For assessment of GRAB_NE_ in HEK293T cells, see Extended Data Fig. 4. For exact *P* values of this and all other figures, see the Supplementary Table. Numerical data for these and all other figures have been deposited in Zenodo (https://doi.org/10.5281/zenodo.20255734).

**Extended Data Fig. 1:**
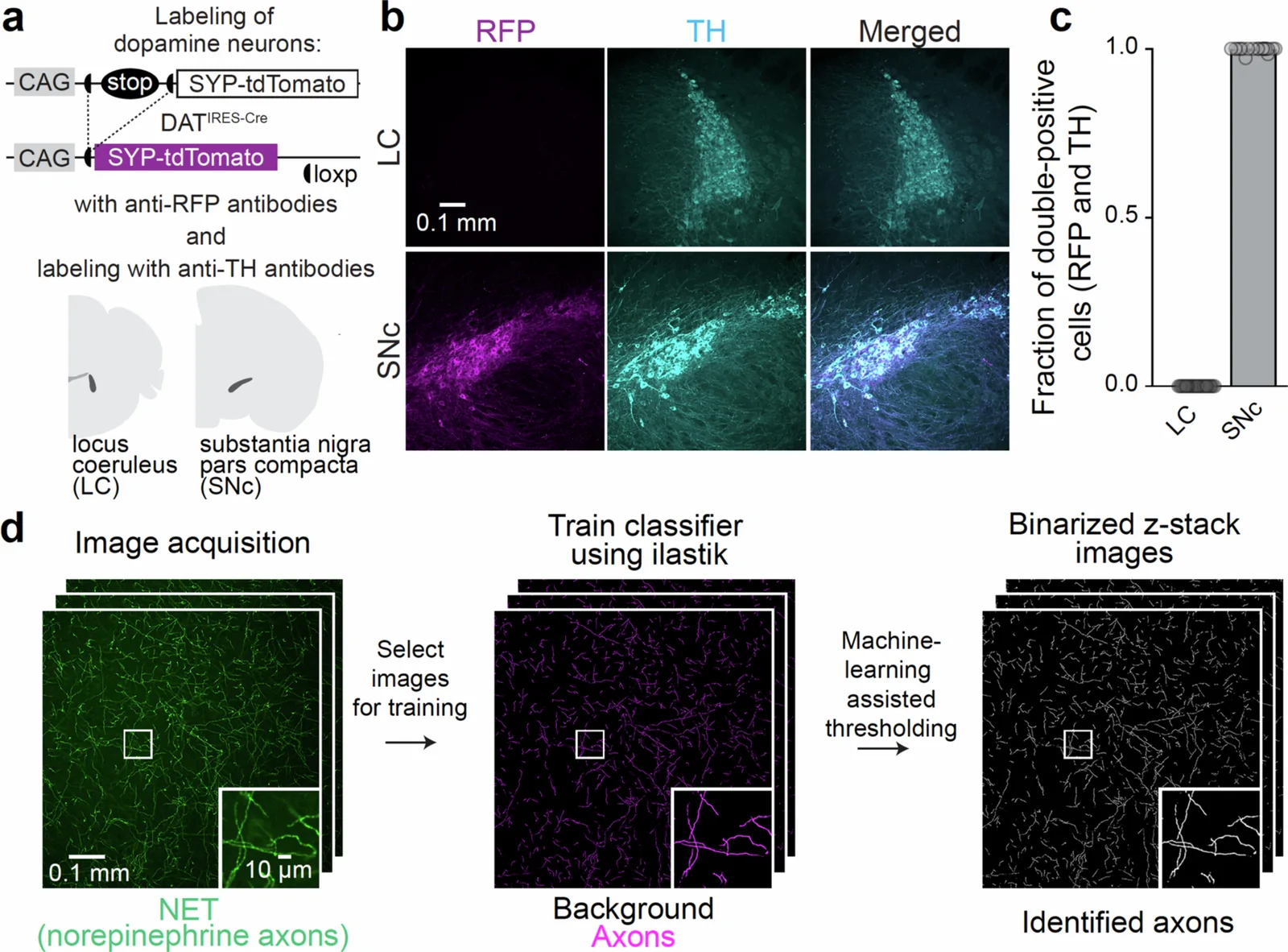
Assessment of Cre-dependent expression of synaptophysin-tdTomato in DAT^IRES-Cre^ mice and workflow for quantification of innervation densities. **a**. Strategy for labeling dopamine and norepinephrine neurons similar to Fig. 1a but with staining for tyrosine hydroxylase (TH) followed by analyses of immunofluorescence in locus coeruleus (LC) and substantia nigra pars compacta (SNc). **b, c**. Representative confocal images of LC and SNc (b) and quantification of the fraction of cells double positive for RFP and TH (c); LC 20 slices from 4 mice, SNc 20/4. On average, 96 ± 6 cells in LC and 88 ± 6 cells in SNc were positive for TH per image. The observation that there are no double-positive cells in LC indicates that the DAT^IRES-Cre^ line does not express Cre in LC neurons. Hence, the strategy of NET labeling combined with RFP-labeling of SYP-tdTomato in DAT^IRES-Cre^ mice can be used to distinguish norepinephrine from dopamine axons. Both norepinephrine and dopamine neurons express TH. **d**. For quantifying innervation densities, z-stacks were acquired on a confocal microscope (left). To generate a classifier that segments axonal signals, background and axonal pixels were manually annotated on a set of individual images of z-stacks using ilastik^40^ (middle). The resulting segmentation classifier was applied to all images in each z-stack to produce binarized z-stacks. Axonal pixels (white) were summated in each binarized image of the z-stack to calculate the total number of stained pixels in the full z-stack (right). Data are mean ± SEM.

**Extended Data Fig. 2:**
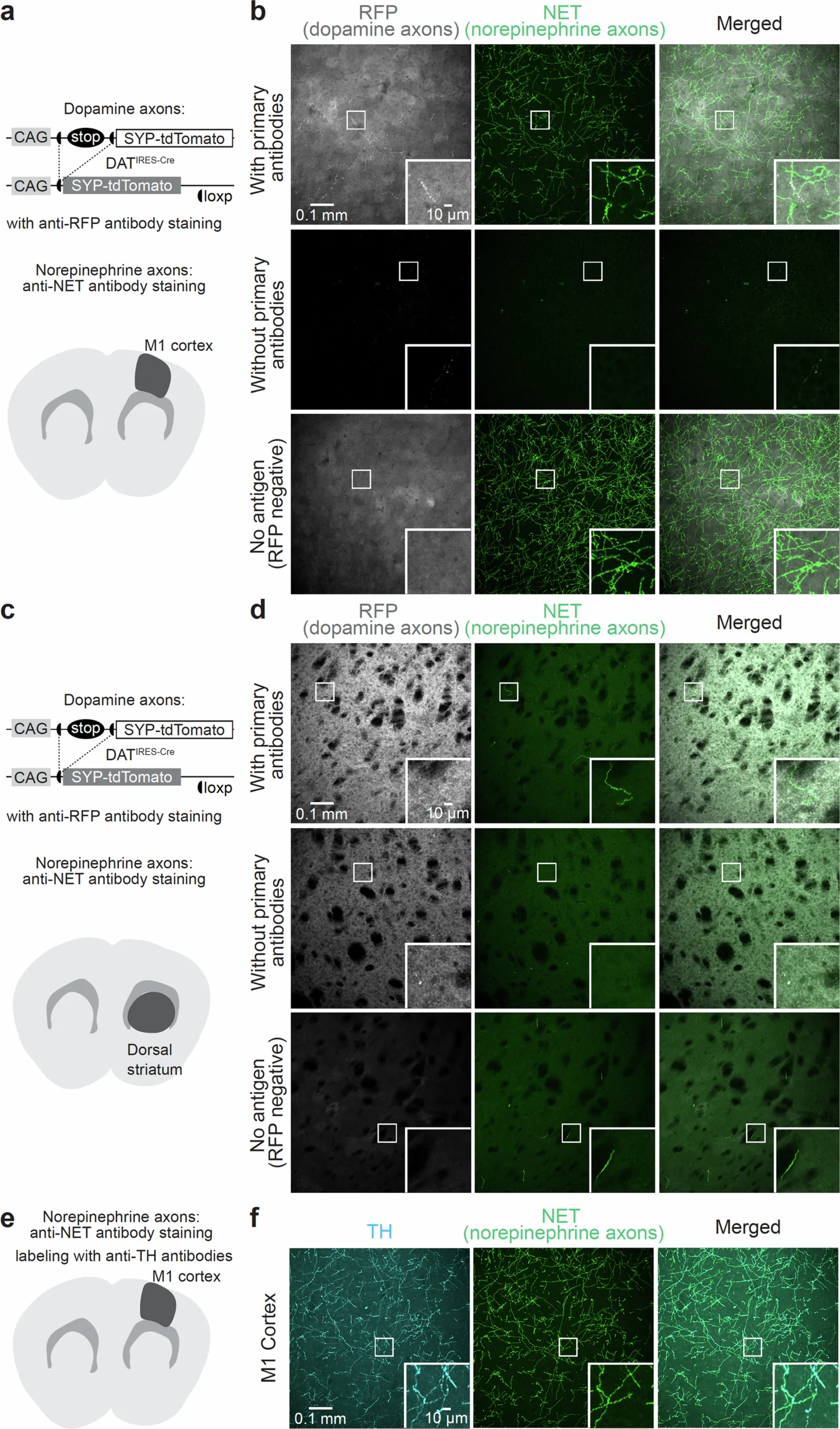
Antibody controls and immunostainings for TH and NET in M1 cortex. **a, b**. Strategy for labeling dopamine and norepinephrine axons in M1 cortex (**a**) and representative confocal images of coronal sections stained with or without primary antibodies and slices not expressing RFP (No antigen) from mice negative for DAT^IRES-Cre^ (**b**). A representative example of 1 slice from 1 mouse is shown per condition. **c, d**. As in **a** and **b**, but in dorsal striatum. A representative example of 1 slice from 1 mouse is shown per condition. **e, f**. Strategy for labeling dopamine and norepinephrine axons in M1 cortex using antibodies against TH and NET (**e**) and representative confocal images of M1 cortex (**f**). Certain dopamine neurons, including some innervating cortex, might not express DAT, and genetic labeling might underestimate cortical dopamine axon density^46,47^. This experiment illustrates that dopamine axons identified as TH^+^/NET^−^ are sparse in M1 cortex, consistent with the genetic labeling strategy. A representative example of 3 slices from 3 mice is shown.

We examined the selectivity of GPCR-based norepinephrine and dopamine sensors^2,4,5^ that are widely used, often in brain areas innervated by both transmitter systems (Supplementary Text). We first quantified the innervation densities of these transmitters in primary motor (M1) cortex and dorsal striatum. Prior work has found dense dopamine innervation in dorsal striatum and a prominent presence of norepinephrine axons in M1 cortex^7–9^. To measure axonal densities, we genetically labeled dopamine axons by Cre-dependent expression of synaptophysin–tdTomato in DAT^IRES-Cre^ mice (Fig. 1a–d and Extended Data Fig. 1a–c). We prepared brain slices containing dorsal striatum and M1 cortex and performed antibody staining against RFP and the norepinephrine transporter (NET) to visualize dopamine and norepinephrine axons, respectively (Fig. 1b–d). Confocal images revealed that in M1 cortex, norepinephrine fibers predominated, and dopamine fibers were sparse. Conversely, in the dorsal striatum, dopamine axons were dense and norepinephrine axons were infrequent. Using a machine learning algorithm for quantification (Extended Data Fig. 1d), we estimate that M1 cortex contains ~90 times more norepinephrine innervation than dorsal striatum (Fig. 1c). By contrast, dopamine axons appear ~500 times denser in striatum than in M1 cortex (Fig. 1d and Extended Data Fig. 2). Assessment of staining in the locus coeruleus (LC) and substantia nigra pars compacta, the corresponding sites of norepinephrine and dopamine neuron somata, confirmed the specificity of the genetic strategy (Extended Data Figs. 1 and 2).

**Extended Data Fig. 3:**
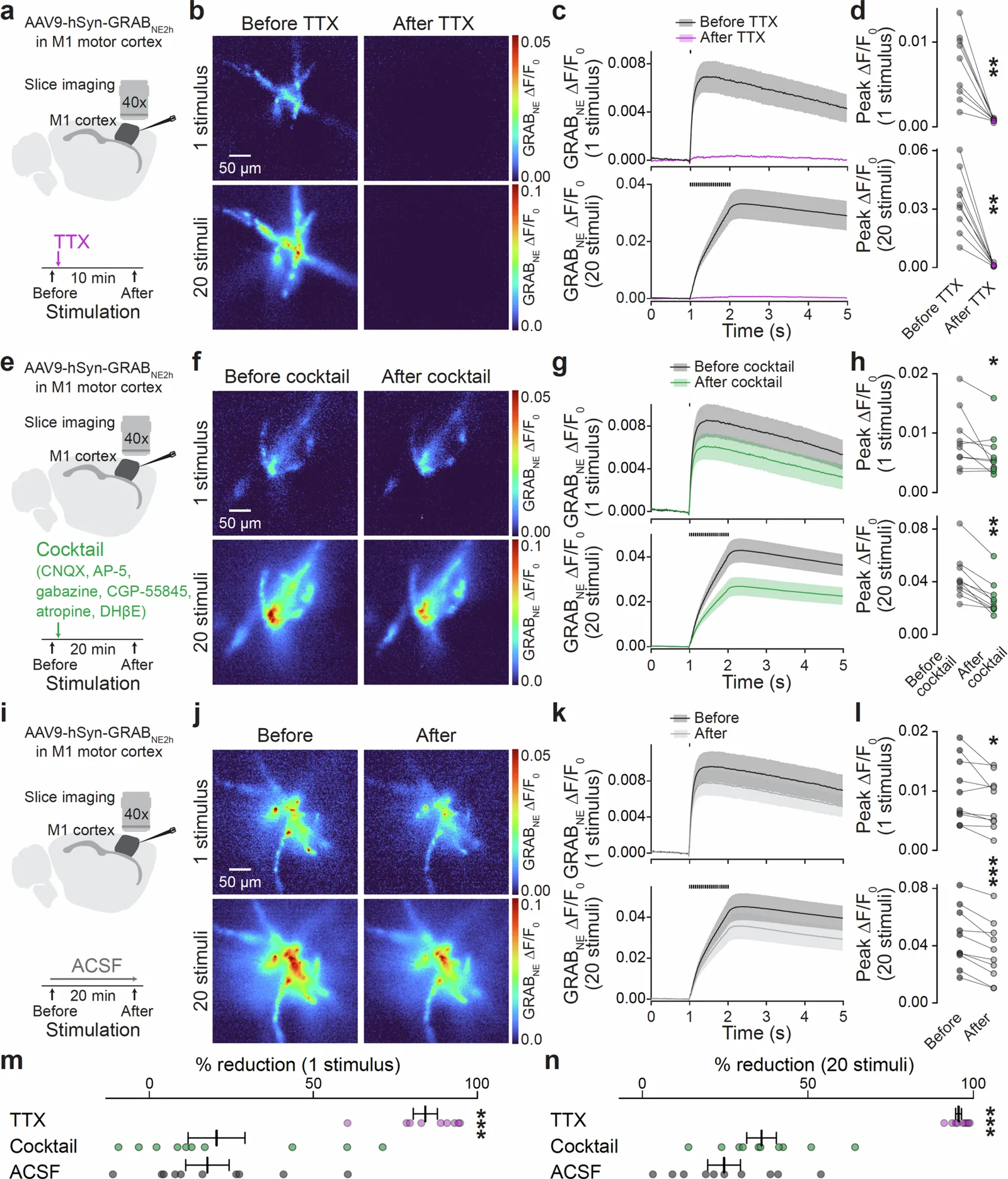
Pharmacological inhibition of GRAB_NE_ transients in brain slices. **a**. Schematic of imaging in cortex before and after 10-minute bath application of the sodium channel blocker tetrodotoxin (TTX, 1 µM) to inhibit action potential firing. **b-d**. Representative images of peak GRAB_NE_ fluorescence in response to 1 stimulus or 20 stimuli at 20 Hz before and after TTX wash-on (**b**) and quantification of GRAB_NE_ ∆F/F_0_ time series (**c**) and peak ∆F/F_0_ (**d**); 9 slices from 3 mice. **e-h**. Same as in **a**-**d**, but with bath application of a cocktail of blockers of AMPA receptors (CNQX, 10 µM), NMDA receptors (D-AP-5, 20 µM), GABA_A_ receptors (gabazine, 10 µM), GABA_B_ receptors (CGP-55845, 300 nM), and muscarinic (atropine, 30 nM) and nicotinic (DhβE, 1 µM) acetylcholine receptors for 20 min; 10/3. **i-l**. Same as in **a**-**d**, but with incubation in ACSF for 20 min; 10/3. **m, n**. Post-hoc assessment of percent reduction in response to 1 stimulus (**m**) or 20 stimuli (**n**) comparing incubation in TTX (**a**-**d**, 10 min), the drug cocktail (**e**-**h**, 20 min), or ACSF (**i**-**l**, 20 min); TTX 9/3, cocktail 10/3, and ACSF 10/3. Overall, rundown during drug cocktail application and incubation in ACSF was similar while TTX strongly inhibited the fluorescence changes. Data are mean ± SEM; *** p < 0.001, ** p < 0.01, * p < 0.05, as assessed by: two-tailed Wilcoxon signed-rank tests in **d** and **h**; two-tailed paired t-tests in **l**; one-way ANOVA and Dunnett’s multiple comparisons post-hoc tests in **m** (compared to ACSF); Kruskal-Wallis and Dunn’s multiple comparisons tests in **n** (compared to ACSF).

To examine the transmitter dynamics resulting from these innervation patterns, we first expressed the norepinephrine sensor GRAB_NE2h_ (α2 adrenergic receptor-based, abbreviated GRAB_NE_; EC_50_ of ~190 nM for norepinephrine and ~9 μM for dopamine)^4^ in M1 cortex or dorsal striatum and analyzed evoked fluorescence transients in acute brain slices with wide-field microscopy (Fig. 1e–k). Single electrical stimuli and stimulus trains (20 stimuli at 20 Hz) yielded fluorescence increases in M1 cortex consistent with norepinephrine innervation (Fig. 1f–h). These transients were fully blocked by inhibition of action potential firing but persisted in a cocktail of blockers for glutamate, GABA and acetylcholine receptors (Extended Data Fig. 3). Single stimuli induced small GRAB_NE_ transients, and 20-stimulus trains produced a buildup with a peak amplitude approximately five times greater than that of a single stimulus (Fig. 1g,h), similar to prior electrochemical studies^8,10,11^. Robust GRAB_NE_ transients were also detected in the dorsal striatum despite the near-absent norepinephrine innervation, although with different properties (Fig. 1i–k). Peak striatal GRAB_NE_ transients were similar between single and train stimuli and decayed more rapidly than the transients in M1 cortex.

We also tested GRAB_DA2m_, a commonly used D2 receptor-based sensor (abbreviated GRAB_DA_; EC_50_ of ~80 nM for dopamine and ~1.2 μM for norepinephrine), which showed response characteristics similar to GRAB_NE_ (Fig. 1l–r). The slower decay of striatal GRAB_DA_ compared to GRAB_NE_ during stimulus trains may reflect differences in ligand affinities, leading to slower GRAB_DA_ off-rates^2,4^. Overall, GRAB_NE_ and GRAB_DA_ signals display similar characteristics when expressed in either M1 cortex or dorsal striatum. However, quantitative comparisons across regions and sensors are difficult to perform. Differences in the biology of the modulatory systems (including innervation densities, secretory machinery and reuptake rates), in sensor properties (including sensor kinetics, affinities, expression levels and cell-surface trafficking) and in experimental conditions (in the present study, including magnification, size of the region of interest and parameters used for quantification) invalidate direct, quantitative cross-comparisons of sensor responses.

**Extended Data Fig. 4:**
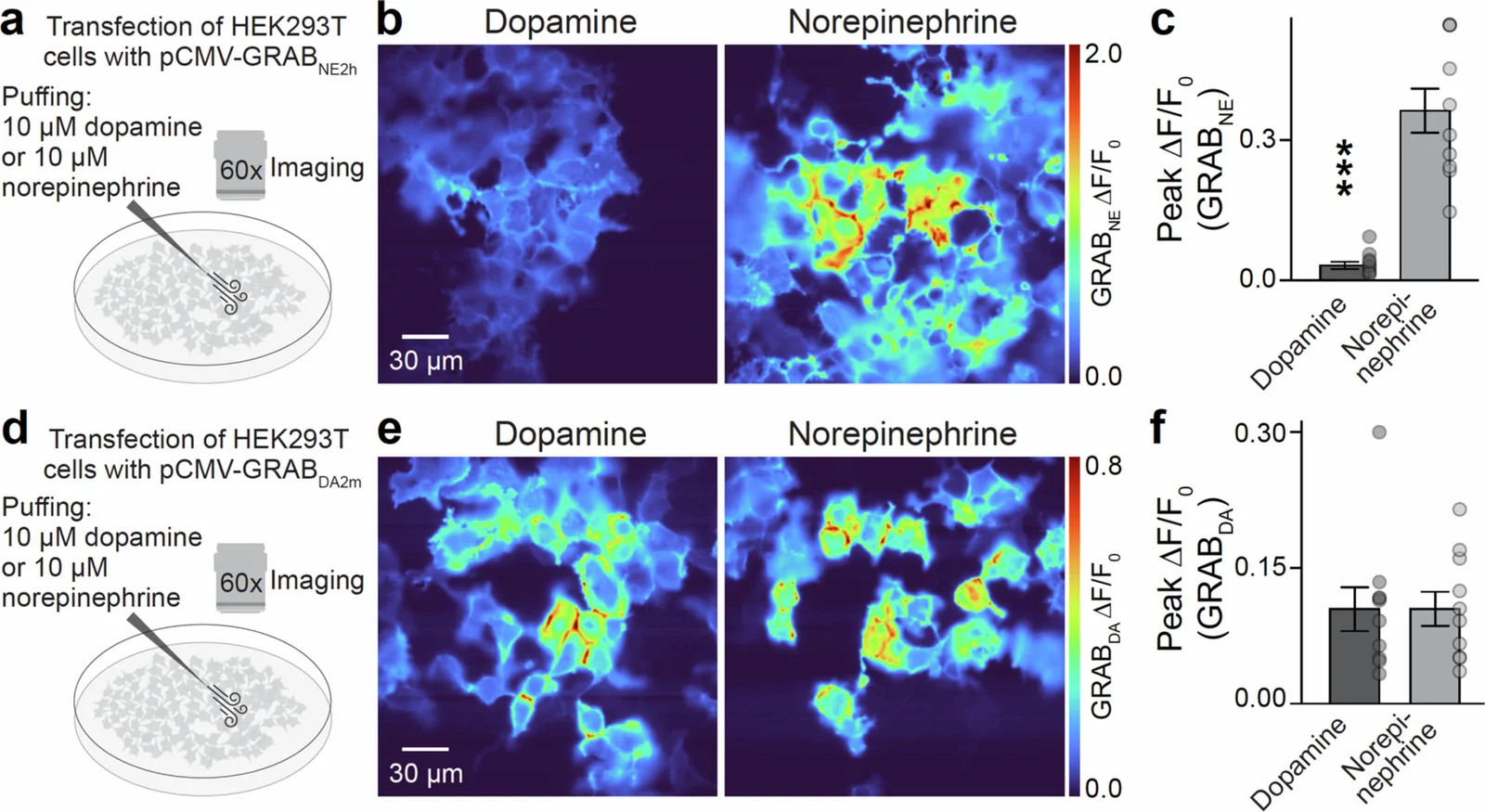
Assessment of GRAB_NE_ and GRAB_DA_ fluorescence in HEK293T cells. **a**. Schematic of the experiment in which 10 µM of dopamine or norepinephrine was puffed onto HEK293T cells transfected with pCMV-GRAB_NE2h_. **b, c**. Representative peak GRAB_NE_ fluorescence after puffing (**b**) and peak ∆F/F_0_ (**c**); 10 coverslips from 3 transfections each. **d-f**. As in **a**-**c**, but with GRAB_DA_; 10/3. These experiments confirm that GRAB_NE_ responds more strongly to 10 µM norepinephrine compared to dopamine, and that GRAB_DA_ responds similarly to both transmitters at this concentration. Sensor response properties tested in vitro are insufficient to predict how a sensor responds in a specific brain area. Data are mean ± SEM; *** p < 0.001, as assessed by two-tailed Mann-Whitney rank-sum test in **c**.

The distinct innervation patterns and sensor signals in M1 cortex and dorsal striatum suggest that GRAB_NE_ might report dopamine in the striatum. This is further supported by the similarity of striatal GRAB_NE_ transients (Fig. 1j,k) to the properties of dopamine release and GRAB_DA_ transients^12–14^. In transfected HEK293T cells, GRAB_NE_ signals were approximately tenfold greater for 10 μM norepinephrine puffs than for 10 μM dopamine puffs (Extended Data Fig. 4a–c). Dopamine-laden vesicles, however, have an intravesicular dopamine concentration of up to ~1 M, and the dopamine concentration at the pore of a fusing vesicle^8,15,16^ far exceeds commonly used concentrations for probing selectivity^1–6^.

**Fig. 2:**
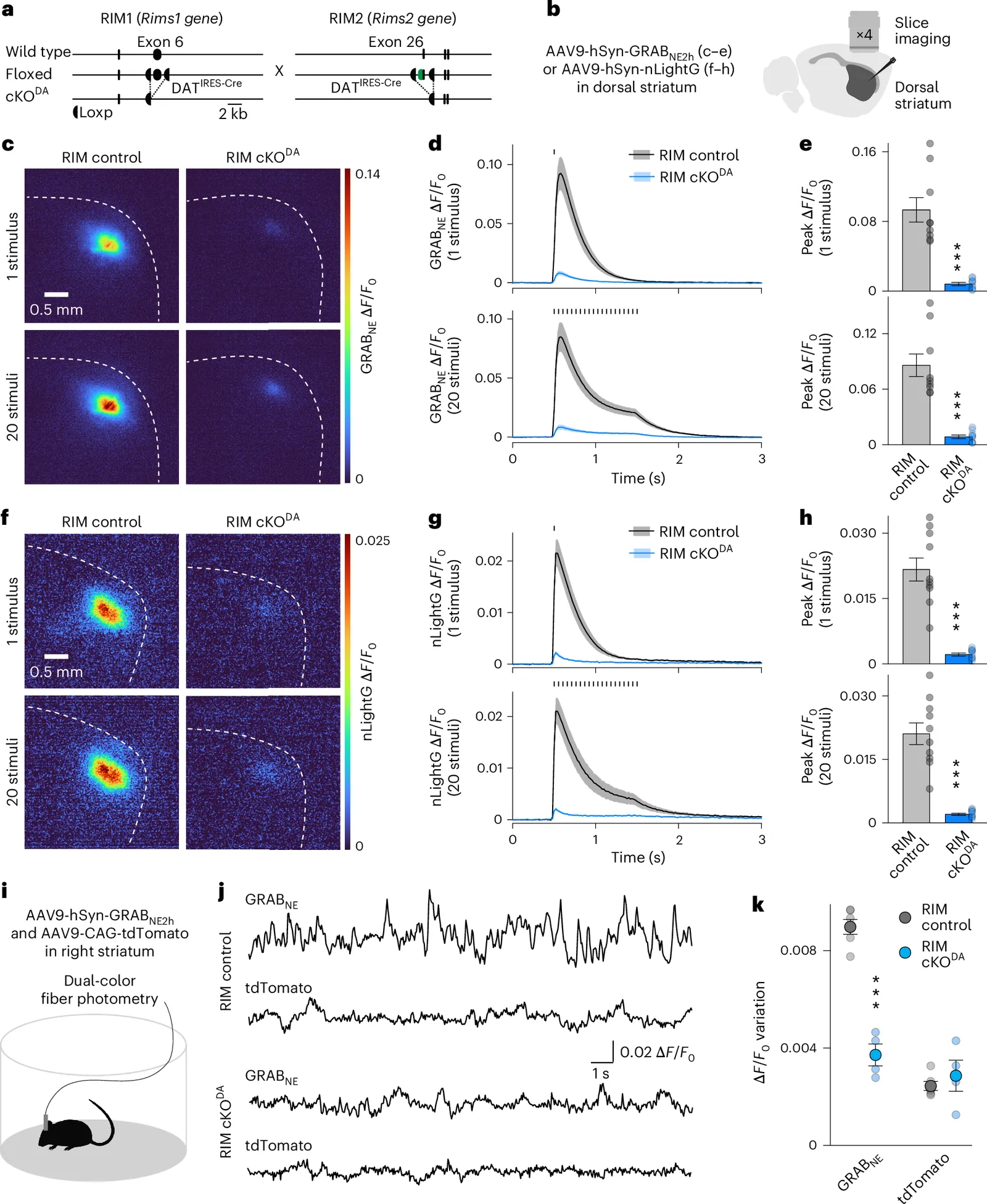
Norepinephrine sensors detect dopamine in dorsal striatum. **a**, Strategy for conditional deletion of RIM1 and RIM2 in dopamine neurons as previously established^12,14,17,18^. **b**, Schematic of slice imaging in dorsal striatum after expressing GRAB_NE_ or nLightG. **c**–**e**, Representative images of peak GRAB_NE_ fluorescence in response to one stimulus or 20 stimuli at 20 Hz (**c**) and quantification of GRAB_NE_ ∆*F*/*F*_0_ time series (**d**) and peak ∆*F*/*F*_0_ (**e**): RIM control, nine slices from three mice; RIM cKO^DA^, nine slices from three mice. **f**–**h**, As in **c**–**e**, but with nLightG instead of GRAB_NE_: RIM control, ten slices from three mice; RIM cKO^DA^, ten slices from three mice. **i**, Schematic of fiber photometry in freely moving mice co-expressing GRAB_NE_ and tdTomato in the right dorsal striatum. **j**,**k**. Representative examples of GRAB_NE_ and tdTomato ∆*F*/*F*_0_ (**j**) and fluorescence variation quantified as the s.d. of GRAB_NE_ and tdTomato ∆*F*/*F*_0_ (**k**): RIM control, six mice; RIM cKO^DA^, four mice. Data are mean ± s.e.m.; ***P < 0.001, as assessed by two-tailed Mann–Whitney rank-sum tests in **e** and **h**, and two-tailed unpaired *t*-test in **k**. For assessment of striatal GRAB_NE_ transients while blocking nicotinic acetylcholine receptors and post hoc assessment of fiberoptic cannula positions, see Extended Data Fig. 5.

**Extended Data Fig. 5:**
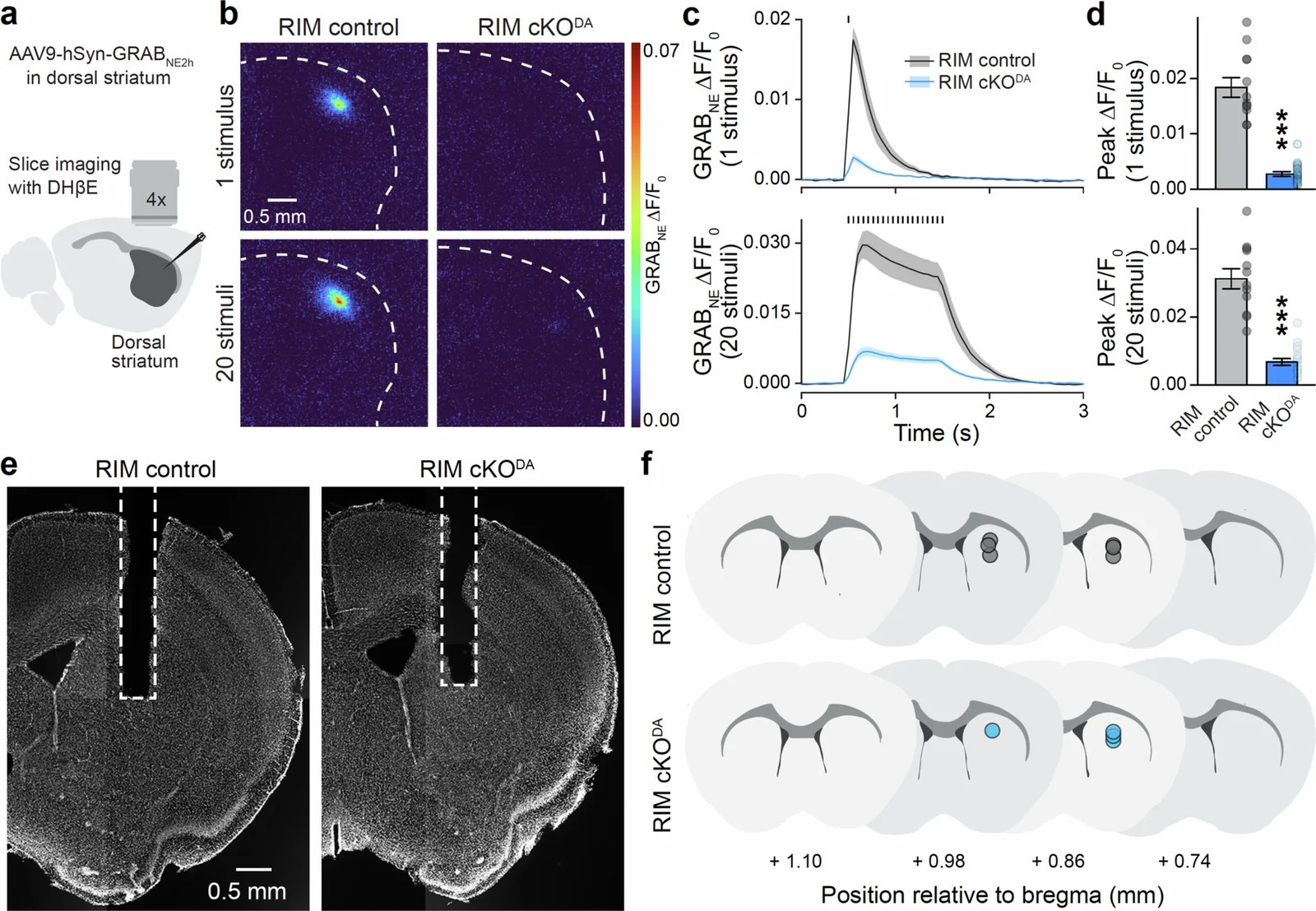
GRAB_NE_ recordings in striatum in the presence of DhβE and assessment of cannula positions for fiber photometry. **a**. Schematic of imaging experiments as in Fig. 2c-e, but in the presence of dihydro-β-erythroidine hydrobromide (DHβE, 1 µM) to block β2-containing nicotinic acetylcholine receptors. **b-d**. Representative images of peak GRAB_NE_ fluorescence in response to 1 stimulus or 20 stimuli at 20 Hz (**b**) and quantification of GRAB_NE_ ∆F/F_0_ time series (**c**) and peak ∆F/F_0_ (**d**); RIM control 12 slices from 3 mice, RIM cKO^DA^ 17/4. **e, f**. Representative images of coronal sections of mice with fiberoptic cannulas (**e**) and illustration of fiberoptic cannula tip positions (**f**) of the 6 RIM control mice and 4 RIM cKO^DA^ mice analyzed in Fig. 2k. Coronal sections were imaged with a slide scanner in DAPI-containing mounting medium, and tip positions were determined relative to a mouse brain atlas^43^ and mapped onto schematics drawn from brain sections. Data are mean ± SEM; *** p < 0.001, as assessed by two-tailed Mann-Whitney rank-sum tests in d.

To directly test whether dopamine induces GRAB_NE_ transients in dorsal striatum, we used mice with conditional deletion of the release site-organizing protein RIM in dopamine neurons (RIM cKO^DA^; Fig. 2a,b). In these mutants, evoked dopamine release is disrupted when assessed with amperometry, whole-cell electrophysiology or GRAB_DA_^12,14,17,18^. Striatal slices from RIM cKO^DA^ mice displayed strong reductions in evoked GRAB_NE_ transients compared to RIM control slices in response to both single and train stimuli (Fig. 2c–e). The same was true when nicotinic acetylcholine receptors, which trigger dopamine release through induction of dopamine axonal action potentials^19–21^, were blocked (Extended Data Fig. 5a–d). We next used nLightG, an alternate GPCR-based norepinephrine sensor engineered from the α1 adrenergic receptor^5^. Like GRAB_NE_, nLightG also reported striatal dopamine release, and RIM cKO^DA^ slices had ~90% reduced nLightG responses (Fig. 2f–h).

To test whether the cross-reactivity is also present in vivo and detected in response to endogenous neural activity, we performed fiber photometric GRAB_NE_ recordings in freely moving RIM control and RIM cKO^DA^ mice (Fig. 2i–k and Extended Data Fig. 5e,f). Fluorescence (*F*) transients were measured as mice explored an open-field arena, and we quantified GRAB_NE_ fluctuations as ∆*F*/*F*_0_, where *F*_0_ is baseline fluorescence. GRAB_NE_ fluctuations were readily detected in RIM control mice, but strongly reduced in RIM cKO^DA^ mice (Fig. 2j,k). This observation is similar to striatal transients measured with dopamine sensors^14^. In summary, these data establish that two commonly used norepinephrine sensors are almost exclusively activated by dopamine in the dorsal striatum.

**Fig. 3:**
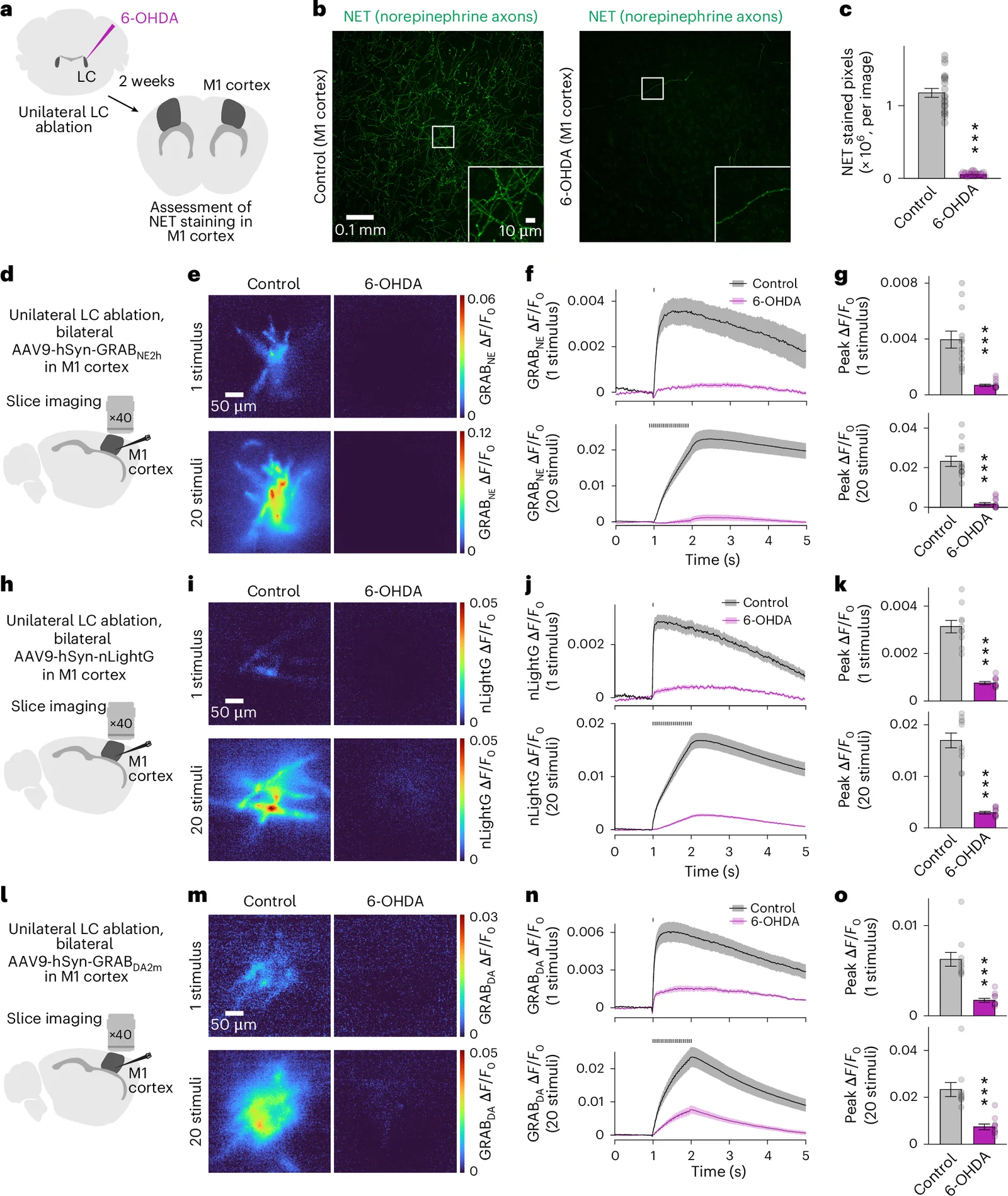
Norepinephrine and dopamine sensors in M1 cortex. **a**, Schematic of unilateral LC ablation with 6-OHDA. Analyses were performed 2 weeks after 6-OHDA injection on confocal images of coronal sections in contralateral (control) and ipsilateral (6-OHDA) hemispheres. Norepinephrine axons were labeled with anti-NET antibodies. **b**,**c**, Representative images of M1 cortex (**b**) and quantification of NET signals (**c**): 20 slices from four mice. **d**. Schematic of bilateral slice imaging in M1 cortex expressing GRAB_NE_ following unilateral ablation of the right LC. **e**–**g**, Representative images of peak GRAB_NE_ fluorescence in response to one stimulus or 20 stimuli at 20 Hz in ipsilateral (6-OHDA) and contralateral (control) hemispheres (**e**) and quantification of GRAB_NE_ ∆*F*/*F*_0_ time series (**f**) and peak ∆*F*/*F*_0_ (**g**): control, 12 slices from three mice; 6-OHDA, 12 slices from three mice. **h**–**k**, As in **d**–**g**, but with nLightG instead of GRAB_NE_: control, ten slices from three mice; 6-OHDA, ten slices from three mice. **l**–**o**, As in **d**–**g**, but with GRAB_DA_ instead of GRAB_NE_: control, ten slices from three mice; 6-OHDA, ten slices from three mice. Data are mean ± s.e.m.; ***P < 0.001, as assessed by two-tailed Mann–Whitney rank-sum tests in **c**, **g**, **k** and **o**. For assessment of GRAB_DA_ in HEK293T cells, see Extended Data Fig. 4. For assessment of histology following unilateral 6-OHDA ablation of LC, see Extended Data Fig. 6. For assessment of GRAB_NE_ responses in hippocampus following unilateral 6-OHDA ablation of LC, see Extended Data Fig. 7. For assessment of GRAB_NE_ responses in striatum, see Extended Data Fig. 8. For assessment of GRAB_NE_ and GRAB_DA_ signals in LC, see Extended Data Fig. 9. For assessment of GRAB_NE_ and GRAB_DA_ signals in mPFC, see Extended Data Fig. 10.

**Extended Data Fig. 6:**
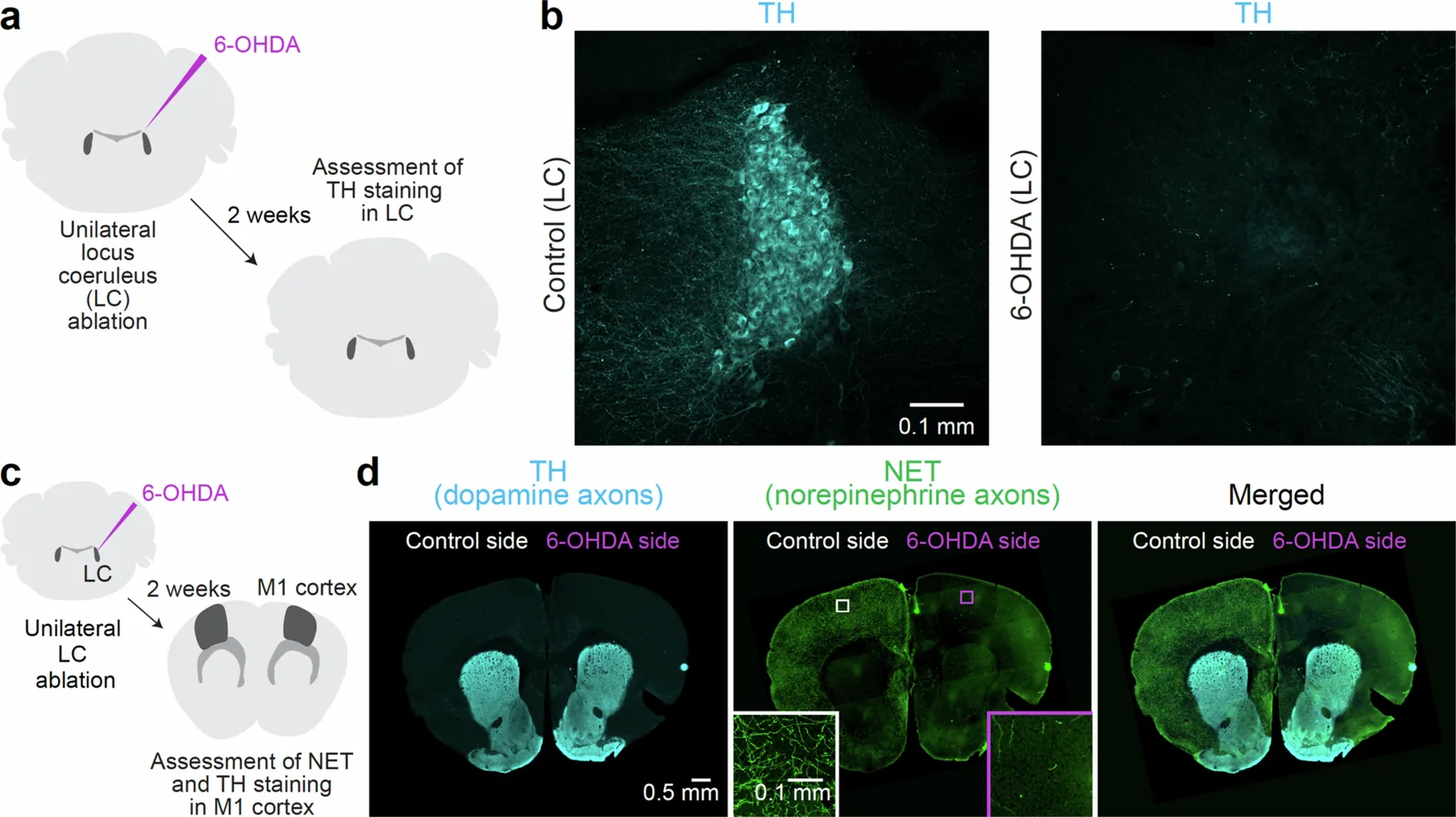
6-OHDA lesion of LC. **a**. Schematic of unilateral 6-OHDA ablation of LC followed by assessment with confocal microscopy. **b**. Confocal images of LC after staining with TH antibodies; a representative image of 4 slices from 4 mice is shown. **c**. Schematic of unilateral 6-OHDA ablation of the right LC. Two weeks after injection, coronal slices were stained against TH and NET, and sections were imaged on a wide-field slide scanning microscope. **d**. Slide scanning images of dorsal striatum and M1 cortex after staining with TH and NET antibodies; a representative image of 3 slices from 3 mice is shown.

**Extended Data Fig. 7:**
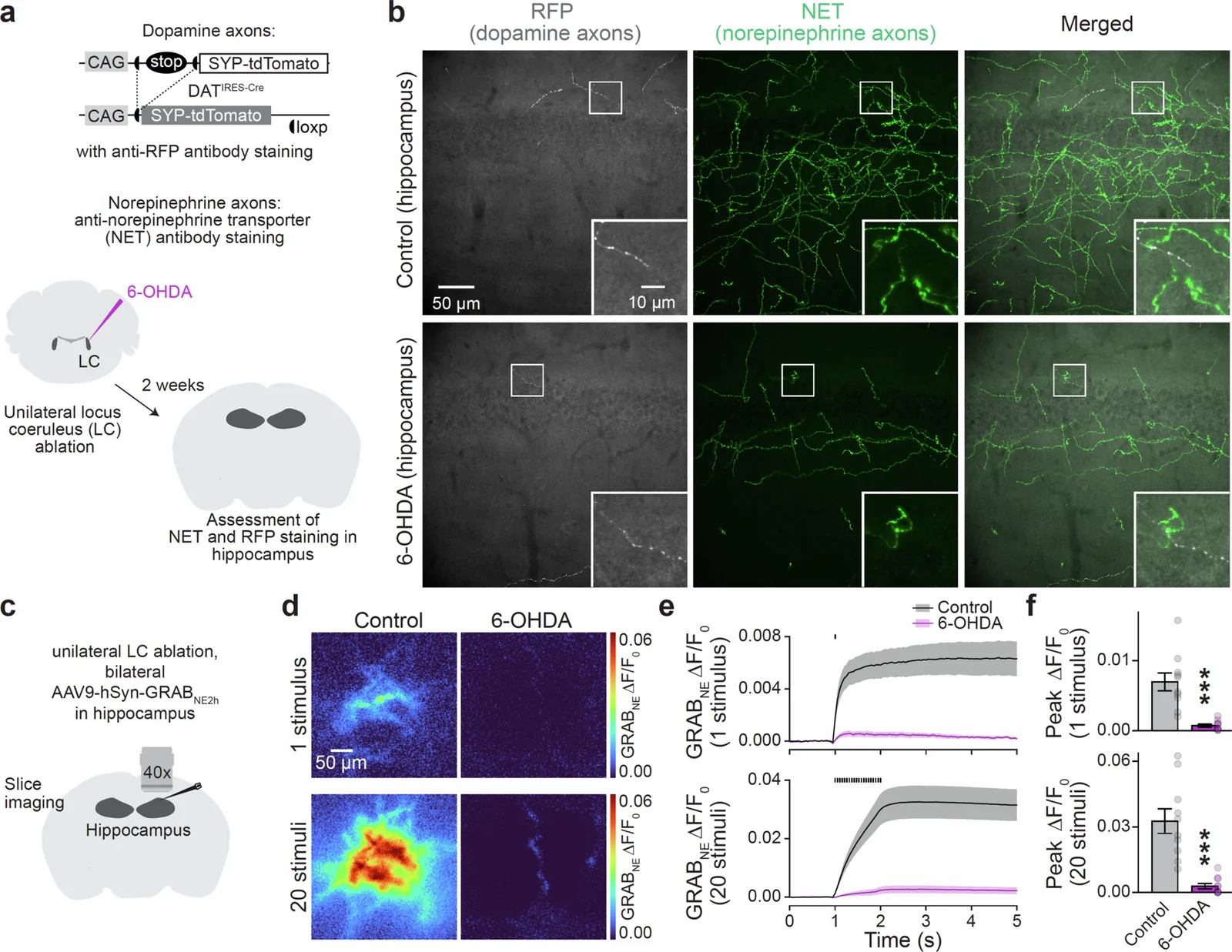
Anatomy of norepinephrine and dopamine axons in hippocampus and GRAB_NE_ responses after 6-OHDA lesion of LC. **a**. Strategy for labeling of dopamine and norepinephrine innervation in the hippocampus following unilateral 6-OHDA ablation of the right LC. **b**. Representative confocal images of hippocampus of 2 slices from 2 mice. **c**. Schematic of bilateral slice imaging in hippocampus expressing GRAB_NE_ following unilateral ablation of the right LC. **d-f**. Representative images of peak GRAB_NE_ fluorescence in response to 1 stimulus or 20 stimuli at 20 Hz in ipsilateral (6-OHDA) and contralateral (control) hemispheres (**d**) and quantification of GRAB_NE_ ∆F/F_0_ time series (**e**) and peak ∆F/F_0_ (**f**); control 10 slices from 3 mice, 6-OHDA 10/3. Data are mean ± SEM; *** p < 0.001, as assessed by two-tailed Mann-Whitney rank-sum tests in f.

**Extended Data Fig. 8:**
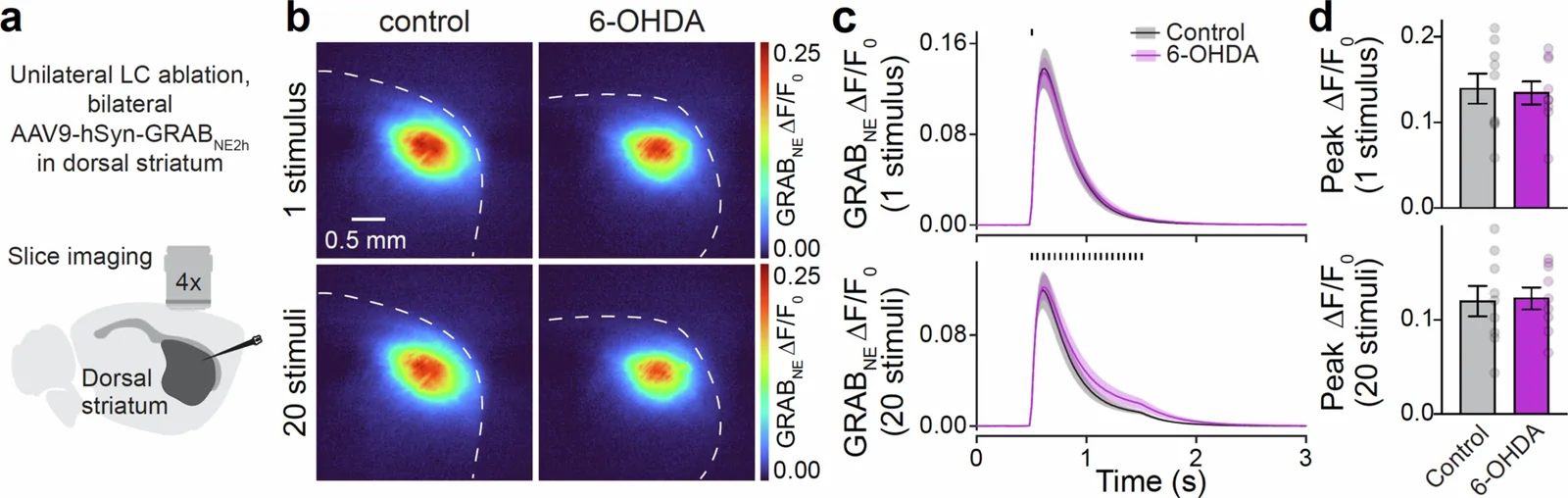
Striatal GRAB_NE_ responses after 6-OHDA lesion of LC. **a**. Schematic of bilateral slice imaging in dorsal striatum expressing GRAB_NE_ with unilateral 6-OHDA ablation of the right LC. **b-d**. Representative images of peak GRAB_NE_ fluorescence in response to 1 stimulus or 20 stimuli at 20 Hz in ipsilateral (6-OHDA) and contralateral (control) hemispheres (**b**) and quantification of GRAB_NE_ ∆F/F_0_ time series (**c**) and peak ∆F/F_0_ (**d**); control 9 slices from 3 mice, 6-OHDA 9/3. Data are mean ± SEM.

Given the robust cortical norepinephrine innervation, we next examined the ability of GRAB_NE_ and nLightG to detect norepinephrine in M1 cortex. We first established that chemical lesion of norepinephrine neurons in the right LC with 6-hydroxydopamine (6-OHDA)^22^ abolished cortical NET staining in the same hemisphere (Fig. 3a–c and Extended Data Fig. 6). We then bilaterally expressed GRAB_NE_ or nLightG in M1 cortex in mice with unilateral LC lesions (Fig. 3d,h). Both sensors responded to single and train stimuli, and in each case, the transients were strongly decreased when the LC was lesioned (Fig. 3d–k). In the hippocampus, where norepinephrine axons dominate, GRAB_NE_ transients were also strongly reduced upon LC lesion (Extended Data Fig. 7), suggesting broad applicability of LC lesion as a control for signal specificity. In contrast to M1 cortex and hippocampus, GRAB_NE_ responses in the striatum were not impaired after LC lesion (Extended Data Fig. 8), indicating that they did not arise from LC neurons and that dopamine neurons were not lesioned by 6-OHDA injection into the LC.

Given the structural similarities of dopamine and norepinephrine, we wondered whether GRAB_DA_ dopamine sensors detect norepinephrine. In transfected HEK293T cells, puffing 10 µM dopamine or norepinephrine produced similar GRAB_DA_ fluorescence changes (Extended Data Fig. 4d–f). We have previously shown that striatal GRAB_DA_ transients are strongly impaired in RIM cKO^DA^ mice^14^, establishing that GRAB_DA_ reports dopamine in the striatum. In M1 cortex, GRAB_DA_ transients induced by single and train stimuli were impaired by ~70% following 6-OHDA lesion of LC (Fig. 3l–o). Hence, GRAB_DA_ responds at least partially to norepinephrine in M1 cortex. The remaining transients are probably a result of cortical dopamine innervation, which may be enhanced upon LC ablation^22^, although cortical dopamine release mechanisms are less understood than those in the striatum and require further study^23^.

**Extended Data Fig. 9:**
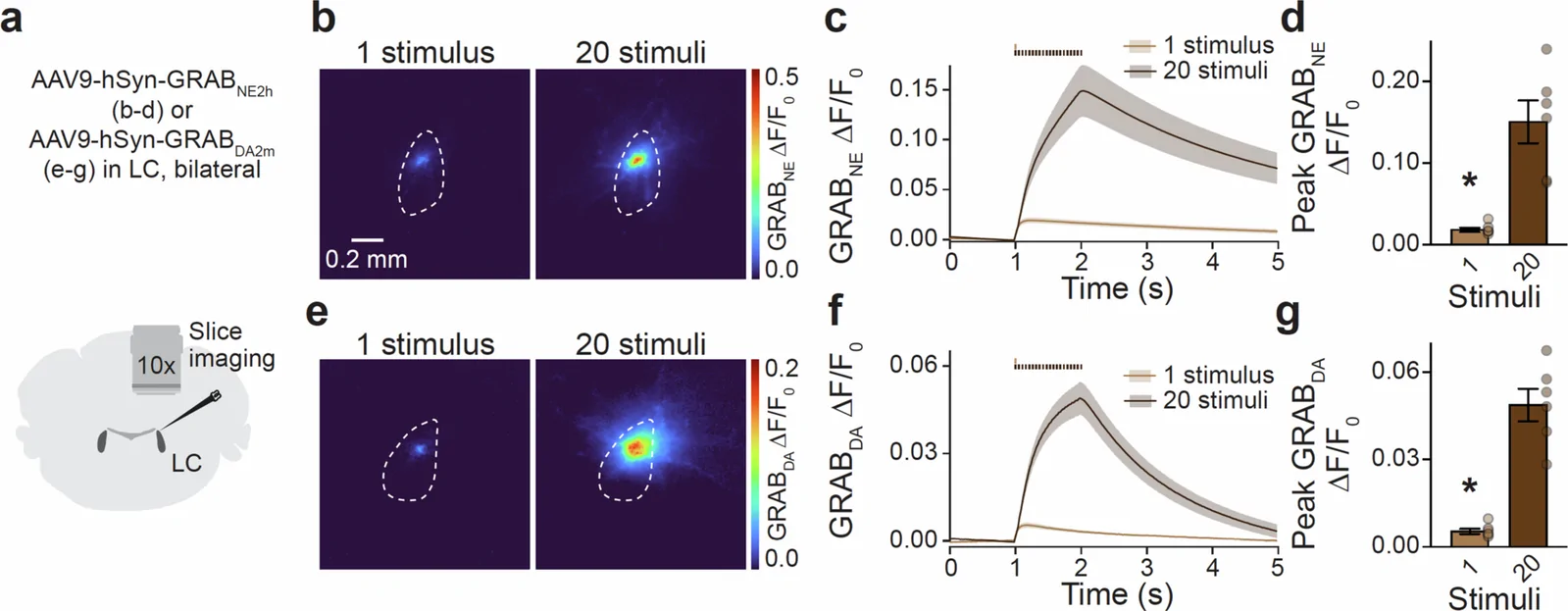
Norepinephrine and dopamine sensors in LC. **a**. Schematic of slice imaging in LC expressing GRAB_NE_ or GRAB_DA_. **b-d**. Representative GRAB_NE_ peak fluorescence in response to 1 stimulus or 20 stimuli at 20 Hz in LC (**b**) and quantification of ∆F/F_0_ time series (**c**) and peak ∆F/F_0_ (**d**); 6 hemispheres from 3 slices from 3 mice. **e-g**. As in **b**-**d**, but with GRAB_DA_; 6/3/3. Data are mean ± SEM; * < 0.05, as assessed by two-tailed Wilcoxon signed-rank tests in d and g.

The LC hardly receives any dopamine innervation (Extended Data Fig. 1b,c)^9^. To evaluate sensor signals in the LC, we expressed either GRAB_NE_ or GRAB_DA_ bilaterally in the LC. Evoked LC GRAB_NE_ transients (Extended Data Fig. 9a–d) had characteristics similar to those measured in cortex (Fig. 1f–h), as did LC GRAB_DA_ transients (Extended Data Fig. 9e–g). With both sensors, single-pulse transients were small compared to those produced by 20 stimuli at 20 Hz, and the transients had prolonged decays. These findings suggest that GRAB_DA_ and GRAB_NE_ report norepinephrine in LC.

**Extended Data Fig. 10:**
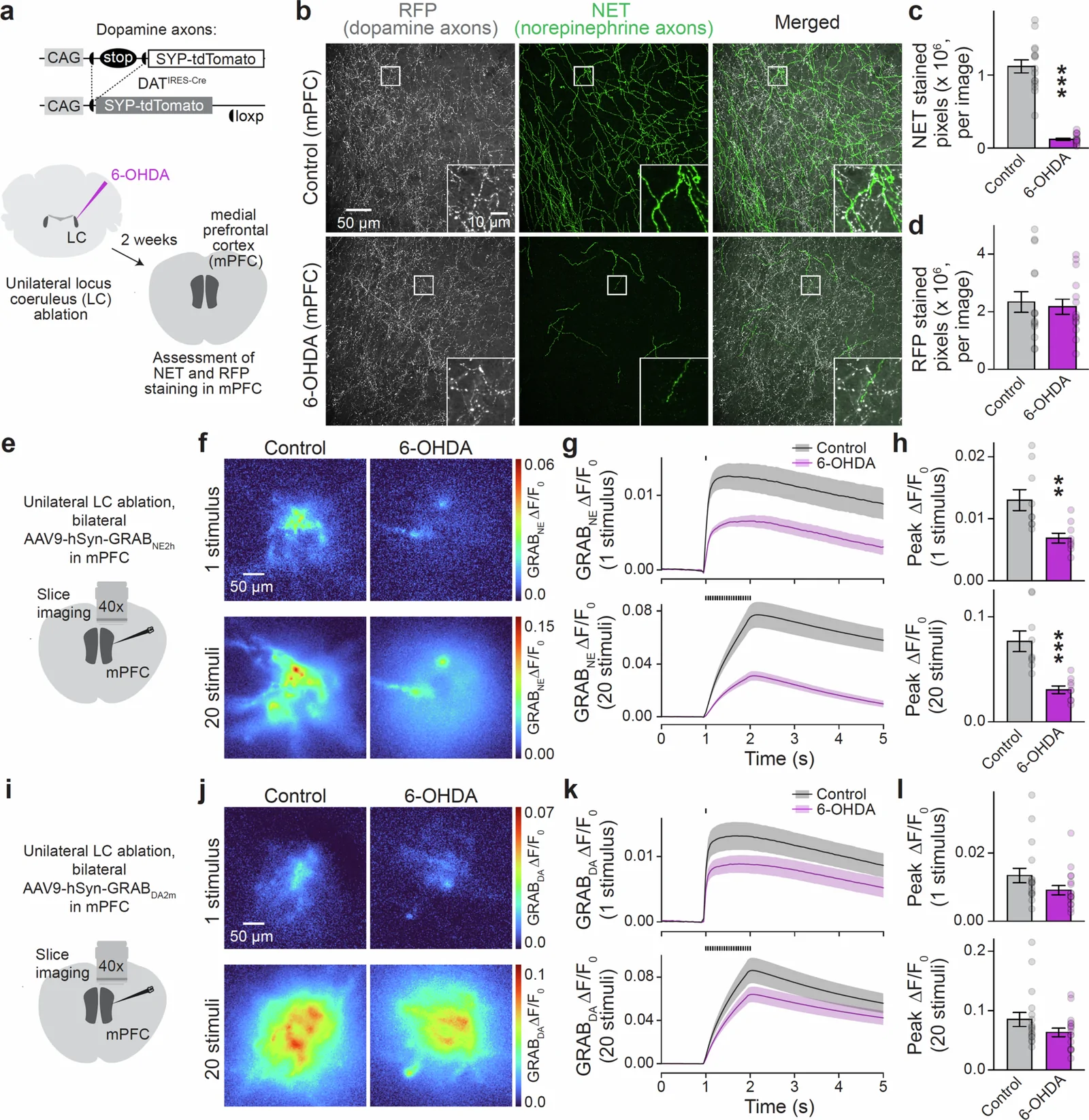
GRAB_NE_ and GRAB_DA_ sensors in medial prefrontal cortex after LC lesion. **a**. Strategy for labeling of dopamine and norepinephrine innervation in the medial prefrontal cortex (mPFC) following unilateral 6-OHDA ablation of LC. Two weeks after injection, dopamine axons were labeled by anti-red fluorescent protein (RFP) antibodies in coronal slices from DAT^IRES-Cre^ mice with Cre-dependent synaptophysin-tdTomato (SYP-tdTomato) expression. Norepinephrine axons were labeled with anti-NET antibodies. Analyses were performed on confocal images taken of both contralateral (control) and ipsilateral (6-OHDA) hemispheres. **b-d**. Representative images (**b**) and quantification of NET (**c**) and RFP (**d**) signals; 15 slices from 3 mice. **e**. Schematic of bilateral slice imaging in mPFC expressing GRAB_NE_ following unilateral 6-OHDA ablation of the right LC. **f-h**. Representative images of peak GRAB_NE_ fluorescence in response to 1 stimulus or 20 stimuli at 20 Hz in ipsilateral (6-OHDA) and contralateral (control) hemispheres (**f**) and quantification of GRAB_NE_ ∆F/F_0_ time series (**g**) and peak ∆F/F_0_ (**h**); control 9 slices from 3 mice, 6-OHDA 9/3. **i-l**. As in **e**-**h**, but with GRAB_DA_ instead of GRAB_NE_; control 17/5, 6-OHDA 17/5. Data are mean ± SEM; *** p < 0.001, ** < 0.01, as assessed by two-tailed Mann-Whitney rank-sum tests in c and h.

Although GRAB_NE_ and GRAB_DA_ show non-selectivity in regions dominated by either dopamine or norepinephrine (Figs. 1–3), it is unclear how the sensors respond in areas with mixed innervation. To address this question, we examined the medial prefrontal cortex (mPFC). Both norepinephrine and dopamine axons robustly innervate the mPFC, and chemical lesioning of the LC with 6-OHDA ablates mPFC norepinephrine fibers, whereas dopamine innervation remains similar in both lesioned and non-lesioned hemispheres (Extended Data Fig. 10a–d). After LC ablation, evoked GRAB_NE_ transients in mPFC are reduced by ~50% (Extended Data Fig. 10e–h). GRAB_DA_ responses display a modest trend towards reductions (Extended Data Fig. 10i–l). These results suggest that GRAB_DA_ may have limited crosstalk in mPFC, but signals in areas with mixed dopamine and norepinephrine innervation are overall difficult to interpret and require the development of control approaches or more specific sensors. As in M1 cortex (Fig. 3n), these mPFC responses have slower kinetics during stimulus trains, different from striatal dopamine (Figs. 1 and 2), possibly because of differences in innervation density, release properties or DAT expression.

The presented results establish that GPCR-based norepinephrine and dopamine sensors (we specifically tested GRAB_NE2h_, nLightG and GRAB_DA2m_) have prominent crosstalk in the brain. Although in vitro affinity measurements reflect sensor properties, innervation densities, which vary dramatically between brain areas (Fig. 1), influence sensor signals. The amount of secreted neurotransmitter is correlated with innervation density, and innervation patterns help predict which transmitter a given sensor reports. In addition, multiple regulatory processes influence extracellular transmitter levels^8,23^. For example, release properties and transmitter buffering, reuptake and degradation shape the local concentration. Ultimately, we find that in the striatum, where dopamine innervation predominates, norepinephrine and dopamine sensors report dopamine release (Figs. 1 and 2). In M1 cortex, norepinephrine axons dominate, and both sensors primarily report norepinephrine secretion (Fig. 3). Therefore, these regions, as well as the hippocampus (Extended Data Fig. 7), are well suited to study the dominant transmitter. Dually innervated areas are more difficult to evaluate. The mPFC, for example, contains balanced dopamine and norepinephrine innervation, and the indicator signals are difficult to interpret (Extended Data Fig. 10) without the development of new specificity controls or sensors.

Our findings are important for many past and ongoing studies (Supplementary Text). Typically, sensor transients are interpreted as reporting the transmitter they were named after, and appropriate loss-of-function experiments are often not performed. A commonly used control to verify sensor activation is pharmacological sensor inhibition, although this approach does not validate transmitter specificity. Crosstalk should always be experimentally addressed, and excluding it within the experimental context of a given study will also be critical for new sensors with different affinities, kinetics or other properties^3,6^. To this end, genetic or chemical silencing is essential to interpret sensor signals in brain slices and in vivo. These manipulations should be done under the specific conditions used in a study and are a necessary standard in the future.

The cross-reactivity we observe for dopamine and norepinephrine sensors probably reflects the high structural similarity of the corresponding transmitters, differing by only one hydroxyl group. Many modulators, including serotonin, other monoamines and more than 100 neuropeptides, operate in the brain^8^, and achieving full signal selectivity may be particularly challenging for transmitters with closely related chemical structures (Supplementary Text). We further note that the electrical stimulation used here induces the simultaneous release of multiple transmitters. In vivo, specific activity patterns govern heterogeneous neurotransmitter dynamics. Examining transmitter-specific release properties by correlating their sensor signals with axonal calcium dynamics or by cell-type-specific optogenetic actuators will help determine features of specific neuromodulators. The spectral overlap between these tools might lead to additional caveats, including artifactual photoactivation, that require appropriate controls^24,25^.

Synthesis and reuptake pathways might further complicate the interpretation of sensor data. Neurons synthesize norepinephrine through enzymatic conversion of dopamine, and LC neurons might co-release both modulators^26,27^. Therefore, incomplete enzymatic conversion can lead to co-release of dopamine and norepinephrine, and each sensor might report mixed signals in response to activities of norepinephrine neurons. The promiscuity of monoamine reuptake transporters may also allow these systems to secrete multiple monoamines^28–30^. As a result, interpreting sensor transients in mixed-innervated regions can be complicated by the uncertain identity of the released transmitter and by sensor non-specificity.

Finally, crosstalk is not a ‘flaw’ of the sensors but instead reflects the properties of GPCRs. Dopamine and adrenergic receptors, for example, can bind both dopamine and norepinephrine^31–33^. Receptors for other neuromodulators also interact with several ligands^34–36^. Although this non-selectivity complicates interpretations of sensor imaging, it underscores that receptor crosstalk probably represents a biologically meaningful mechanism to convey information and to regulate cells, circuits and behavior.

## Methods

### Mice

DAT^IRES-Cre^ mice (RRID:IMSR_JAX:006660, B6.SJL-*Slc6a3*^*tm1.1(cre)Bkmn*^/J)^37^ were bred to mice containing a CAG promoter-driven *loxP*-STOP-*loxP*-synaptophysin–tdTomato cassette (SYP–tdTomato) in the *Rosa26* locus (RRID:IMSR_JAX:012570, B6;129S-*Gt(ROSA)26Sor*^*tm34.1(CAG-Syp/tdTomato)Hze*^/J) as previously established^12^. Mice carrying floxed alleles for RIM1 expressed by the *Rims1* gene (RRID:IMSR_JAX:015832, *Rims1*^*tm3Sud*^/J)^38^ and RIM2 expressed by the *Rims2* gene (RRID:IMSR_JAX:015833, *Rims2*^*tm1.1Sud*^/J)^39^ were bred to DAT^IRES-Cre^ mice^37^ as previously established^12,14,17,18^. RIM cKO^DA^ mice were homozygous for floxed RIM1 and RIM2 and heterozygous for DAT^IRES-Cre^. RIM control mice were heterozygous for both floxed RIM alleles and DAT^IRES-Cre^ and were either littermates of RIM cKO^DA^ mice or age-matched non-littermates from intercrosses of the same alleles. Mice used for non-mutant comparisons were either wild-type mice or contained floxed RIM1 and RIM2 alleles but were negative for DAT^IRES-Cre^. Male and female mice were included in all experiments regardless of sex. Intracranial injections for viral expression were performed at 5–12 weeks of age, and functional and morphological analyses were performed at 8–17 weeks of age. Mice were maintained on a mixed background (containing C57BL/6 and 129/Sv) and housed in a room set to 20–24 °C and 50% humidity. Mice used for fiber photometry were housed in a reversed light–dark cycle, and experiments were performed during the dark phases. Genotype comparisons were performed by an experimenter blind to genotype during data acquisition and analyses. Experiments were performed following approved protocols of the Harvard University Institutional Animal Care and Use Committee.

### Confocal image acquisition and processing

Morphological analyses were adapted from protocols used previously^12,14,17,18^. Mice were deeply anesthetized with 5% isoflurane and transcardially perfused with 30 ml of PBS followed by 40 ml of 4% paraformaldehyde (PFA) in PBS. Brains were post-fixed in 4% PFA in PBS for 24 h and stored in PBS until sectioning. Mice with stereotaxic 6-OHDA injections in the LC were perfused 2 weeks following the injection. Brain slices (50 µm thick) were prepared in ice-cold PBS using a vibratome. Antigen retrieval was performed for 30 min at 60 °C in a solution containing 150 mM NaCl, 10 mM Tris base, 1 mM EDTA and 0.05% Tween 20 (pH 9.0). Slices were then permeabilized in PBS containing 0.25% Triton X-100 (PBST) in three consecutive 10-min incubations at room temperature (20–24 ºC) and blocked in PBST containing 10% normal goat serum for 2 h at room temperature. Staining with primary antibodies was performed for 24 h at 4 °C in PBST with 10% normal goat serum. Sections were washed three times for 10 min in PBST, followed by secondary antibody staining for 2 h at room temperature in PBST with 10% normal goat serum. After another three 10-min washes, the slices were mounted on glass slides for imaging. The primary antibodies used were mouse IgG1 anti-NET (1:1,000; Synaptic Systems, 260 011, RRID:AB_2636907, lab antibody code A265), guinea pig anti-RFP (1:1,000; Synaptic Systems, 390 004, RRID:AB_2737052, A258) and rabbit anti-TH (1:2,000; Millipore, AB152, RRID:AB_390204, A66). Secondary antibodies used were Alexa Fluor 405 goat anti-rabbit (1:500; Thermo Fisher Scientific, A-31556, RRID:AB_221605, lab antibody code S1), Alexa Fluor 488 goat anti-mouse IgG1 (1:500; Thermo Fisher Scientific, A-21121, RRID:AB_2535764, S7) and Alexa Fluor 568 goat anti-guinea pig (1:500; Thermo Fisher Scientific, A-11075, RRID:AB_2534119, S27). Confocal images of M1 cortex, dorsal striatum, substantia nigra pars compacta and LC were captured on an inverted spinning disk confocal microscope with a ×20, 0.8 numerical aperture air objective (Figs. 1 and 3 and Extended Data Figs. 1 and 6). A total of 30–50 images were acquired in *z* from each section at a 0.9 µm step size; 20 contiguous planes were manually selected for analysis. Confocal images of mPFC and hippocampus were captured using a ×40, 0.75 numerical aperture air objective (Extended Data Figs. 7 and 10); 22–43 images were acquired in *z* from each section at a 0.9 µm step size, and ten contiguous planes were manually selected for analysis. Whole-section images containing M1 cortex and striatum were acquired using a brightfield slide scanning microscope with a ×4, 0.16 numerical aperture air objective (Extended Data Fig. 5e) or a ×10, 0.6 numerical aperture air objective (Extended Data Fig. 6d). For confocal imaging and slide scanning, identical acquisition settings were used within a specific channel for an entire experiment. To quantify axonal innervation (Figs. 1c,d and 3c and Extended Data Fig. 10c,d), ilastik^40^ was used to generate classifiers that automatically identified axonal structures and produced binary masks for image analyses (Extended Data Fig. 1d). A classifier was trained for each channel in each experiment to distinguish foreground (axons) and background pixels. Classifiers were trained in the ‘pixel classification’ workflow. Either full *z*-stacks or selected representative regions of *z*-stacks were selected to train each classifier, and at least one *z*-stack per mouse was selected for training. The classifier was gradually trained by sequential manual labeling of sets of foreground and background pixels. Once a classifier was generated, it was applied to the entire *z*-stack of the same channel in an experiment, and the resulting masked images were exported. Individual masks were visually checked by an experimenter for accuracy before proceeding. The number of positive pixels in each image plane was then summed using Python to quantify the total signal. To calculate the number of double-positive RFP and tyrosine hydroxylase somata in the LC and substantia nigra pars compacta (Extended Data Fig. 1), 20-plane maximum projection *z*-stack images were created, and each channel’s brightness and contrast were identically adjusted across images. Double-positive somata were then manually counted. Representative images for figures were processed using Fiji and are composed of 20-plane maximum intensity projection *z*-stacks. In Fig. 1b, brightness and contrast in the green (NET) channel were adjusted identically in M1 cortex and striatum, and brightness and contrast in the gray (RFP) channel were differentially adjusted to render cortical dopamine axons visible and to prevent saturation of striatal dopamine axons. For representative images, brightness and contrast were adjusted linearly and identically across conditions.

### GRAB_NE_ and GRAB_DA_ experiments in HEK293T cells

HEK293T cells were cultured as previously established^41,42^. Specifically, cells acquired from ATCC (CRL-3216, RRID:CVCL_0063; mycoplasma-free, human cell line of female origin) were expanded and stored as frozen stocks until use. After thawing, the cells were grown in filtered DMEM supplemented with 10% FBS (Atlas Biologicals, F-0500-D) and 1% penicillin–streptomycin. HEK293T cells were split every 1–3 days at a ratio of 1:3 to 1:10. Cell batches were replaced after ~20 passages by thawing a fresh vial from the expanded stock. For puffing experiments, HEK293T cells were plated on 0.1 mm-thick Matrigel-coated glass coverslips (12 mm) at ~10–20% confluency in 24-well plates. After ~24 h, cells were transfected with the calcium phosphate method at ~50–60% confluency with 350 ng of pDisplay-GRAB_NE2h_-IRES-mCherry-CAAX (abbreviated pCMV-GRAB_NE2h_; lab plasmid code p1109; Addgene, 208691)^4^ or 200 ng of pDisplay-GRAB_DA2m_-IRES-mCherry-CAAX (abbreviated pCMV-GRAB_DA2m_; p1110)^2^. Between 24 h and 48 h after transfection, individual coverslips were transferred to a recording chamber perfused with a recording solution containing 126 mM NaCl, 2.5 mM KCl, 2 mM CaCl_2_, 1.3 mM MgSO_4_, 1 mM NaH_2_PO_4_, 12 mM glucose and 26.2 mM NaHCO_3_ at 33–35 °C with a flow rate of 2–3 ml min^−1^. The recording solution was continuously bubbled with 95% O_2_ and 5% CO_2_. Fluorescence imaging was performed using an Olympus BX51WI epifluorescence microscope. Fluorescence signals were excited with a 470 nm LED and digitized with a scientific complementary metal-oxide-semiconductor camera (sCMOS, Hamamatsu Orca-Flash4.0). Data were acquired with a ×60, 0.9 numerical aperture water-immersion objective at 512 × 512 pixels per frame and at 20 frames per second, with an exposure time of 50 ms. Dopamine hydrochloride or noradrenaline bitartrate was dissolved in Milli-Q H_2_O to generate 100 mM stocks. These stocks were then diluted into recording solution to generate 10 µM working solutions and puffed for a 3-s epoch onto the cells using a pulled glass pipette (3–5 µm tip diameter) connected to a Picospritzer. Analyses of ∆*F*/*F*_0_ responses were performed in Python. The *F* value for each time point encompasses the averaged pixel intensities of the entire 512 × 512 image. *F*_0_ represents the averaged fluorescence values captured during the 1.5 s window before puffing. ∆*F*/*F*_0_ was calculated as (*F* − *F*_0_) / *F*_0_ for each time point to generate time series plots. Peak ∆*F*/*F*_0_ values were then extracted from the time series after puffing and plotted. To generate representative heatmaps, peak fluorescence for each pixel was averaged from a 3.75 s window following the puff. ∆*F*/*F*_0_ was then calculated for each pixel and plotted in the heatmap images. The resulting images were smoothed with a Gaussian blur (σ = 1).

### Stereotaxic surgeries

Stereotaxic surgeries were performed following previously established methodology^12,14,18,20^. Mice were anesthetized with 5% isoflurane and mounted on a stereotaxic frame, and 1.5–2% isoflurane was used to maintain stable anesthesia. Following exposure of the skull, a hand drill was used to create a small burr hole for syringe placement. For unilateral and bilateral injections in the dorsal striatum (coordinates relative to bregma: 1.00 mm anterior, ±2.00 mm lateral, 2.50 mm below the dura), adeno-associated viruses (AAVs) were delivered using a syringe coupled to a microinjector pump at a volume of 1 µl with a flow rate of 100 nl min^−1^. Viruses were diluted to a working titer of 10^11^ copies per ml before injection. Mice used for fiber photometry were unilaterally equipped with fiberoptic cannulas (400 µm diameter) in the right dorsal striatum that were placed immediately following AAV delivery (coordinates relative to bregma: 1.0 mm anterior, 2.0 mm lateral, 2.3 mm below the dura) and fixed using quick adhesive dental cement. For unilateral and bilateral viral injections in M1 cortex (coordinates relative to bregma: 1.00 mm anterior, ±1.40 mm lateral, 1.25 mm below the dura), mPFC (coordinates relative to bregma: 2.0 mm anterior, ±0.5 mm lateral, 2.5 mm below the dura), hippocampus (coordinates relative to bregma: 1.60 mm posterior, ±1.35 mm lateral, 1.90 mm below the dura) and the LC (coordinates relative to bregma: 5.4 mm posterior, ±1.0 mm lateral, 3.3 mm below the dura), manually pulled borosilicate glass pipettes (tip diameter, 3–5 µm) were used for AAV delivery. A volume of 500 nl was delivered at a flow rate of 50 nl min^−1^ in M1 cortex, mPFC and hippocampus. For bilateral AAV expression targeted to the LC, 1 µl of virus was injected at a flow rate of 100 nl min^−1^ on each side. For 6-OHDA injections, 1 µl of 6-OHDA (10 µg µl^−1^ in PBS containing 0.01% ascorbic acid) was delivered to the right LC (coordinates relative to bregma: 5.40 mm posterior, 0.75 mm lateral, 3.30 mm below the dura) at a flow rate of 100 nl min^−1^ with a manually pulled borosilicate glass pipette (tip diameter, 3–5 µm). Following injections, microinjectors were left in place for 10 min to allow the injected volume to settle. Mice were treated for post-surgical pain and were returned to their home cages before experiments.

### AAVs

AAVs were of the AAV9 serotype, as defined by the capsid. Serotypes are reported in the figures independent of pseudotyping, and AAVs are available from Biohippo, BrainVTA, WZBioscience and Addgene. AAV9-hSyn-GRAB_DA2m_ (stocks at 1–2 × 10^12^ copies per ml; Biohippo, BHV12400556-9) and AAV9-hSyn-GRAB_NE2h_ (stocks at 1–2 × 10^12^ copies per ml; Biohippo, BHV12400441-9) were purchased. AAV9-hSyn-nLightG (stocks at 1.4 × 10^12^ copies per ml; AAV2/9.hSyn.nLightG) was produced by the Viral Vector Facility of the University and ETH Zürich. For photometry, AAV9-hSyn-GRAB_NE2h_ was co-injected with AAV9-CAG-tdTomato (stocks at 1–2 × 10^13^ copies per ml; Addgene, 59462-AAV9).

### Slice imaging and analyses

Imaging in acute brain slices was performed as previously established^14,20^. Specifically, mice were deeply anesthetized with isoflurane and decapitated. The brain was removed, and 250 μm-thick brain slices were cut using a vibratome in ice-cold cutting solution containing 75 mM NaCl, 2.5 mM KCl, 7.5 mM MgSO_4_, 75 mM sucrose, 1 mM NaH_2_PO_4_, 12 mM glucose, 26.2 mM NaHCO_3_, 1 mM myo-inositol, 3 mM sodium pyruvate and 1 mM sodium ascorbate. Sagittal slices were prepared for imaging in M1 cortex or dorsal striatum, and coronal slices were cut for imaging in the LC. For experiments involving 6-OHDA ablation of the LC, sagittal slices from both hemispheres were cut for imaging in M1 cortex or dorsal striatum, and coronal sections from both hemispheres were cut for imaging in mPFC and hippocampus. After cutting, slices were incubated for 30 min at 37 °C in a recovery solution composed of 126 mM NaCl, 2.5 mM KCl, 2 mM CaCl_2_, 1.3 mM MgSO_4_, 1 mM NaH_2_PO_4_, 12 mM glucose, 26.2 mM NaHCO_3_, 1 mM myo-inositol, 3 mM sodium pyruvate and 1 mM sodium ascorbate. Slices were then incubated for a minimum of 30 min at room temperature. Imaging was performed in a chamber continuously perfused with artificial cerebral spinal fluid (ACSF) containing 126 mM NaCl, 2.5 mM KCl, 2 mM CaCl_2_, 1.3 mM MgSO_4_, 1 mM NaH_2_PO_4_, 12 mM glucose and 26.2 mM NaHCO_3_ at 33–35 °C with a flow rate of 2–3 ml min^−1^. Solutions were constantly bubbled with 95% O_2_ and 5% CO_2_, and data acquisition was completed within 5 h of slicing. For Extended Data Figs. 5, 1 µM of dihydro-β-erythroidine hydrobromide (DHβE; diluted from 100 mM stock) was included in the recording solution. Fluorescence imaging was performed with an Olympus BX51WI epifluorescence microscope. A 470 nm LED was used for excitation, and the measured signals were digitized with a sCMOS camera (Hamamatsu Orca-Flash4.0). A ×4, 0.13 numerical aperture air objective was used for striatal imaging. LC imaging was performed with a ×10, 0.30 numerical aperture water-immersion objective. Imaging in M1 cortex, mPFC and hippocampus was performed with a ×40, 0.80 numerical aperture water-immersion objective. Electrical stimulation was applied using a pulled glass pipette filled with ACSF (tip diameter, 3–5 µm, 0.5–1 MΩ). Single stimuli and 20-stimulus 20 Hz trains were delivered at an intensity of 90 µA for two to three replicates with 2 min interstimulus intervals using a linear stimulus isolator. A biphasic wave (0.25 ms in each phase) was applied for each stimulus. For Extended Data Fig. 3a–d, fluorescence changes in response to single and train stimuli were assessed before and after wash-on of tetrodotoxin (1 µM) for 10 min. For Extended Data Fig. 3e–h, fluorescence changes in response to single and train stimuli were assessed before and after wash-on of a cocktail containing 6-cyano-7-nitroquinoxaline-2,3-dione (CNQX; 10 µM), D-AP-5 (20 µM), SR-95531 hydrobromide (gabazine; 10 µM), CGP-55845 hydrochloride (300 nM), atropine (30 nM) and DHβE (1 µM) for 20 min. For Extended Data Fig. 3i–l, fluorescence changes in response to single and train stimuli were assessed before and after incubation in ACSF for 20 min. Fluorescence images were acquired at 512 × 512 pixels per frame and 50 frames per second with an exposure time of 20 ms. Striatal slice imaging using DHβE and slice imaging performed in the mPFC and hippocampus were performed at 20 frames per second with an exposure time of 50 ms per frame. Image analyses were performed in Python. For quantification of transients in the dorsal striatum and LC (Figs. 1 and 2 and Extended Data Figs. 5, 8 and 9), circular regions of interest (ROIs) with 30–50-pixel radii were manually selected in each image time series to calculate time series of ∆*F*/*F*_0_. The position of each ROI was centered around the visually identified peak signal. The radii of manually selected ROIs were constant for each brain area at 50 pixels for LC and 30 pixels for dorsal striatum. For recordings in M1 cortex shown in Fig. 1 and Extended Data Fig. 3, ROIs were generated from the top 10% of responding pixels in the first 20-stimuli replicate. The resulting mask was then used to calculate ∆*F*/*F*_0_ in the remaining replicates. Imaging of M1 cortex, mPFC and hippocampus in Fig. 3 and Extended Data Figs. 7 and 10 was analyzed using manually selected circular ROIs with a radius of 100 pixels. For recordings in M1 cortex, mPFC, hippocampus, LC and dorsal striatum with nLightG or in the presence of DHβE, bleach correction was performed on raw signals with Python’s SciPy curve-fitting module using an exponential decay function. To determine *F*_0_, 500 ms windows before the onset of electrical stimulation were averaged within an ROI. The *F* value for each time point represents the averaged pixel intensities within each ROI. ∆*F*/*F*_0_ was then calculated as (*F* − *F*_0_) / *F*_0_ for each time point and averaged to generate the time series plots. Peak ∆*F*/*F*_0_ values were then extracted from the time series and plotted. This process was also used to create example heat plot images for the time window of 280–500 ms after stimulation. The resulting images were smoothed with a Gaussian blur (σ = 1) to generate the example heat plot images for display in the figures. Recordings from control and experimental conditions were processed identically.

### Fiber photometry

Fiber photometric recordings in freely moving mice were performed as previously established^14,20^. At 21 days after surgical implantation, the fiberoptic cannula was connected to an optic patch cord, and the mice were allowed to freely roam for 1-h epochs in a circular arena (43.1 cm diameter, 35.6 cm high) illuminated by infrared light (850 nm, 30 μW cm^−2^ as measured from the arena surface). Silicon photodiodes were used to convert fluorescence to electrical signals, which were then amplified by photodiode amplifiers and collected by a multifunction I/O card at 10,000 Hz. LEDs (470 nm and 565 nm; Thorlabs) were used to excite the channels and applied at 157 μW and 17 μW, respectively, as previously established^14^. Output power was chosen to hold the raw fluorescence signals for the green and red channels in a similar range, and excitation for each LED was applied in an alternating pattern at 25 Hz. During the 40 ms active period, each excitation channel was on for 10 ms, and the average output of each channel was assessed and set according to previously established protocols^20^. For analyses, the raw photometry signal *F* was first processed with a low-pass filter at 0.01 Hz to estimate *F*_0_, and Δ*F*/*F*_0_ was calculated as (*F* − *F*_0_) / *F*_0_ and analyzed from the first 30 min of the 1-h data acquisition. To quantify the fluorescence fluctuations, a 640 ms sliding window was applied to Δ*F*/*F*_0_ transients to calculate an average of the standard deviation. Brief illuminations (200 ms light pulses, 565 nm LED light source, 50 μW cm^−2^ measured at the bottom of the arena) were applied at random time intervals ranging from 60 to 210 s during the 1-h epoch. At the end of the experiment, mice were deeply anesthetized with 5% isoflurane and transcardially perfused with 30 ml PBS followed by 40 ml of 4% PFA in PBS. Brains were then extracted and post-fixed in 4% PFA in PBS for 24 h following perfusion. Brain slices were cut at 50 µm in ice-cold PBS on a vibratome and mounted on glass slides with a medium containing DAPI. Images were acquired using a slide scanning microscope with a ×4, 0.16 numerical aperture air objective. Cannula positions were determined with respect to a mouse brain atlas^43^ and are displayed in Extended Data Fig. 5 on schematics drawn from corresponding brain sections.

### Statistics and reproducibility

Data are shown as mean ± s.e.m., with asterisks indicating significance (*P < 0.05, **P < 0.01, ***P < 0.001). Data were plotted using Python (Python Software Foundation, 3.11.4), MATLAB (MathWorks R2025b) and/or Prism (GraphPad 10.6.1), and individual data points are included in each figure whenever possible. The number of observations in each experiment was based on previous publications^12,14,20,44^ and on trial experiments; no statistical methods were used to predetermine sample sizes. For each experiment, the definitions of sample size are specified in the figure legends, and sample sizes for each plot are included in each figure legend. For genotype comparisons, the experimenter was blind to genotype while acquiring and analyzing data. All data that met quality standards described throughout the methods were included, and there was no exclusion of outliers for genotype comparisons or comparisons of experimental conditions. Experimental groups and data were not randomized, but sorted by genotype or condition after they were analyzed. Datasets were tested for normality with Anderson–Darling, D’Agostino–Pearson omnibus, Shapiro–Wilk and Kolmogorov–Smirnov tests. Datasets were tested for variance with the *F-*test for two-group comparisons and Brown–Forsythe and Bartlett’s tests for three-group comparisons. Datasets that met criteria for normalcy and equal variance across all tests were analyzed using parametric statistical tests; non-parametric tests were used otherwise. The specific statistical tests used are described in each figure legend, *P* value ranges are shown in each figure and exact *P* values of statistical tests are tabulated in the Supplementary Table.

### Reporting summary

Further information on research design is available in the Nature Portfolio Reporting Summary linked to this article.

## Online content

Any methods, additional references, Nature Portfolio reporting summaries, source data, extended data, supplementary information, acknowledgements, peer review information; details of author contributions and competing interests; and statements of data and code availability are available at https://doi.org/10.1038/s41593-026-02379-w.

## Supplementary information


Supplementary InformationSupplementary Discussion, Supplementary Table.
Reporting Summary


## Data Availability

Data points generated for this study are included in the figures whenever possible. Numerical data presented in figures are available on Zenodo (https://doi.org/10.5281/zenodo.20255734)^45^. Additional data are available from the corresponding author upon request.
