# Supplementary material for: Norepinephrine and dopamine sensor crosstalk depends on local innervation density
Source: Nat Neurosci. 2026 Aug 5;29(9):2103–9. doi: 10.1038/s41593-026-02379-w (PMC13533832; doi:10.1038/s41593-026-02379-w)
Supplement: Supplementary file 1 [file 41593_2026_2379_MOESM1_ESM.pdf]

nature neuroscience

Supplementary information https://doi.org/10.1038/s41593-026-02379-w

Norepinephrine and dopamine sensor crosstalk depends on local innervation density

In the format provided by the authors and unedited

Supplementary information guide

The supplementary information contains a supplementary discussion and a supplementary table. The text describes the previous use of GPCR-based sensors for dopamine and norepinephrine in a variety of rodent brain areas and contains additional references. The text also describes the challenge of sensor crosstalk for other transmitter systems. The supplementary table lists statistical tests and p-values for each figure.

Supplementary text. Previous studies with GPCR-based sensors in brain areas with dual dopamine and norepinephrine innervation and the challenge of sensor crosstalk

In the present study, we test whether GPCR-based dopamine and norepinephrine sensors are specific for their corresponding transmitters in the brain. These sensors have been widely used in brain areas that are innervated by both norepinephrine and dopamine neurons.

Dopamine and norepinephrine sensors are susceptible to crosstalk given the structural similarities of their respective ligands and of the endogenous GPCRs used for sensor development[48]. The two transmitters differ by a single hydroxyl group, and their receptors have similar structures in their binding pockets[49,50]. Hence, intrinsic features of these modulatory systems are the basis of sensor crosstalk.

Previous rodent studies have used norepinephrine or dopamine sensors in the cortex[3,4,51–76] , in the hippocampus[5,77–83], and in the cerebellum[84], brain areas in which norepinephrine innervation is generally higher in density compared to dopamine innervation[9,85–88]. Areas with dopamine and norepinephrine innervation densities that are likely in a similar range[89–92] have also been studied with these sensors, for example the basolateral amygdala[3,6,60,93–95], the lateral septum[96], the lateral habenula[97], the lateral hypothalamus[4,5,80,98,99], the basal forebrain[100], and the thalamus[101,102]. Finally, dually innervated brain areas in which dopamine dominates over norepinephrine[90,92,103] have been characterized with the sensors as well, for example the striatum[1–3,14,60,61,99,104–120] and the midbrain[60,120].

Overall, the sensor signals in these brain regions might be due to either dopamine or norepinephrine release. Without cell type- or projection-specific manipulations to remove either dopamine or norepinephrine innervation and/or secretion, it is difficult to conclude which

transmitter is detected in dually innervated brain areas when GPCR-based sensors for dopamine or norepinephrine are used.

These challenges in the interpretation of sensor fluorescence are not limited to dopamine and norepinephrine GPCR-based indicators. Carbon nanotube-based near infrared catecholamine nanosensors (nIRCats), for example, detect both dopamine and norepinephrine[121]. In addition, many transmitters, peptides, and metabolites act throughout the brain, and some bear structural similarities. Dopamine and norepinephrine are part of the larger monoamine family, which also includes epinephrine, serotonin, histamine, and melatonin. Because of the structural similarities of these transmitters and their receptors, crosstalk of the corresponding sensors may occur. Another specific example is the crosstalk between oxytocin and vasopressin, two functionally overlapping neuropeptides, with oxytocin sensor-activation by vasopressin[36,122]. The excitatory neurotransmitter glutamate is structurally similar to glycine and aspartate, and some glutamate sensors are activated by these chemicals[123,124]. Taken together, our and other studies underscore that crosstalk may apply to a variety of sensors and should always be considered in experimental design and interpretation.

References

48. Wu, Y., Zeng, L. & Zhao, S. Ligands of Adrenergic Receptors: A Structural Point of View. *Biomolecules* **11**, (2021).
49. Zhuang, Y. *et al.* Structural insights into the human D1 and D2 dopamine receptor signaling complexes. *Cell* **184**, 931-942.e18 (2021).
50. Xu, J. *et al.* Structural insights into ligand recognition, activation, and signaling of the α2A adrenergic receptor. *Sci. Adv.* **8**, eabj5347 (2022).
51. Basu, A. *et al.* Frontal Norepinephrine Represents a Threat Prediction Error Under Uncertainty. *Biol. Psychiatry* **96**, 256–267 (2024).
52. Glaeser-Khan, S. *et al.* Spatiotemporal Organization of Prefrontal Norepinephrine Influences Neuronal Activity. *eNeuro* **11**, (2024).
53. Kjaerby, C. *et al.* Memory-enhancing properties of sleep depend on the oscillatory amplitude of norepinephrine. *Nat. Neurosci.* **25**, 1059–1070 (2022).
54. Ma, C. *et al.* Microglia regulate sleep through calcium-dependent modulation of norepinephrine transmission. *Nat. Neurosci.* **27**, 249–258 (2024).
55. Pittolo, S. *et al.* Dopamine activates astrocytes in prefrontal cortex via α1-adrenergic receptors. *Cell Rep.* **40**, (2022).
56. Saito, A. *et al.* Social defeat stress enhances the rewarding effects of cocaine through α1A adrenoceptors in the medial prefrontal cortex of mice. *Neuropharmacology* **242**, (2024).
57. Yang, J.-H. *et al.* Frontal cortex norepinephrine, serotonin, and dopamine dynamics in an innate fear-reward behavioral model. *bioRxiv* doi:10.1101/2023.11.27.568929 (2023).
58. Breton-Provencher, V., Drummond, G. T., Feng, J., Li, Y. & Sur, M. Spatiotemporal dynamics of noradrenaline during learned behaviour. *Nature* **606**, 732–738 (2022).
59. Patriarchi, T. *et al.* Ultrafast neuronal imaging of dopamine dynamics with designed genetically encoded sensors. *Science* **360**, eaat4422 (2018).
60. Grove, J. C. R. *et al.* Dopamine subsystems that track internal states. *Nature* **608**, 374–380 (2022).
61. Labouesse, M. A. *et al.* A chemogenetic approach for dopamine imaging with tunable sensitivity. *Nat. Commun.* **15**, (2024).
62. Yuan, Z. *et al.* Optimized deep brain stimulation for anterior cingulate cortex inhibition produces antidepressant-like effects in mice. *Neuron* **113**, 3363-3373.e4 (2025).
63. Inácio, A. R. *et al.* Brain-wide presynaptic networks of functionally distinct cortical neurons. *Nature* **641**, 162–172 (2025).
64. Wang, H. *et al.* Frontal noradrenergic and cholinergic transients exhibit distinct spatiotemporal dynamics during competitive decision-making. *Sci. Adv.* **11**, eadr9916 (2025).
65. Piantadosi, S. C. *et al.* An integrated microfluidic and fluorescence platform for probing in vivo neuropharmacology. *Neuron* **113**, 1491-1506.e6 (2025).
66. Huang, L., Chang, Y., Yang, Z., Lynch, W. J. & Venton, B. J. Coding principles of dopaminergic transmission modes. *Sci. Adv.* **11**, eadx6367 (2025).
67. Deng, Z., Fei, X., Zhang, S. & Xu, M. A time window for memory consolidation during NREM sleep revealed by cAMP oscillation. *Neuron* **113**, 1983-1997.e7 (2025).
68. Liu, Y. A. *et al.* Phase synchrony between prefrontal noradrenergic and cholinergic signals indexes inhibitory control. *Nat. Commun.* **16**, 7260 (2025).
69. Zeidler, Z. *et al.* Dopaminergic projections to the prefrontal cortex are critical for rapid threat avoidance learning. *Curr. Biol.* **35**, 4259-4269.e3 (2025).
70. Chen, J. & Fontanini, A. Cortical dopaminergic signaling mediates planning of directional movements. *Sci. Adv.* **11**, eadt2730 (2025).

71. Bae, J. W., Yi, J. H., Choe, S. Y., Li, Y. & Jung, M. W. Cortical VIP neurons as a critical node for dopamine actions. *Sci. Adv.* **11**, eadn3221 (2025).
72. Luskin, A. T. *et al.* Heterogeneous pericoerulear neurons tune arousal and exploratory behaviours. *Nature* **643**, 437–447 (2025).
73. Yogesh, B., Heindorf, M., Jordan, R. & Keller, G. B. Quantification of the effect of hemodynamic occlusion in two-photon imaging of mouse cortex. *Elife* **14**, (2025).
74. Hjort, M. M. *et al.* Prefrontal to ventral tegmental area dynamics drive contingency degradation. *Nature* doi:10.1038/s41586-026-10443-5 (2026).
75. Guo, L. *et al.* Impact of NPAS2 on mPFC dopamine synthesis and nap behavior. *Nat. Commun.* **17**, (2026).
76. Darmohray, D. *et al.* Brainstem circuit for sickness-induced sleep. *Sci. Adv.* **11**, eady0245 (2025).
77. Privitera, M. *et al.* Noradrenaline release from the locus coeruleus shapes stress-induced hippocampal gene expression. *Elife* **12**, (2024).
78. Wilmot, J. H. *et al.* Phasic locus coeruleus activity enhances trace fear conditioning by increasing dopamine release in the hippocampus. *Elife* **12**, (2024).
79. Wilson, L. R. *et al.* Partial or Complete Loss of Norepinephrine Differentially Alters Contextual Fear and Catecholamine Release Dynamics in Hippocampal CA1. *Biological psychiatry global open science* **4**, 51–60 (2023).
80. Feng, J. *et al.* A Genetically Encoded Fluorescent Sensor for Rapid and Specific In Vivo Detection of Norepinephrine. *Neuron* **102**, 745-761.e8 (2019).
81. Matarasso, A. *et al.* Stimulus-Dependent Dopamine Dynamics from Locus Coeruleus Axons. *bioRxiv* doi:10.1101/2025.09.15.676390 (2025).
82. Godino, A. *et al.* Dopamine D1-D2 signalling in hippocampus arbitrates approach and avoidance. *Nature* **643**, 448–457 (2025).
83. Zhang, E. T., Saglimbeni, G. S., Feng, J., Li, Y. & Bruchas, M. R. Dentate gyrus norepinephrine ramping facilitates aversive contextual processing. *Nat. Commun.* **16**, 454 (2025).
84. Canton-Josh, J. E., Qin, J., Salvo, J. & Kozorovitskiy, Y. Dopaminergic regulation of vestibulo-cerebellar circuits through unipolar brush cells. *Elife* **11**, (2022).
85. Plummer, N. W. *et al.* An Intersectional Viral-Genetic Method for Fluorescent Tracing of Axon Collaterals Reveals Details of Noradrenergic Locus Coeruleus Structure. *eNeuro* **7**, (2020).
86. Verney, C. *et al.* Morphological evidence for a dopaminergic terminal field in the hippocampal formation of young and adult rat. *Neuroscience* **14**, 1039–52 (1985).
87. Kempadoo, K. A., Mosharov, E. V, Choi, S. J., Sulzer, D. & Kandel, E. R. Dopamine release from the locus coeruleus to the dorsal hippocampus promotes spatial learning and memory. *Proc. Natl. Acad. Sci. U. S. A.* **113**, 14835–14840 (2016).
88. Tsetsenis, T. *et al.* Midbrain dopaminergic innervation of the hippocampus is sufficient to modulate formation of aversive memories. *Proc. Natl. Acad. Sci. U. S. A.* **118**, (2021).
89. Korchynska, S. *et al.* A hypothalamic dopamine locus for psychostimulant-induced hyperlocomotion in mice. *Nat. Commun.* **13**, (2022).
90. Lerner, T. N. *et al.* Intact-Brain Analyses Reveal Distinct Information Carried by SNc Dopamine Subcircuits. *Cell* **162**, 635–647 (2015).
91. Lutas, A., Fernando, K., Zhang, S. X., Sambangi, A. & Andermann, M. L. History-dependent dopamine release increases cAMP levels in most basal amygdala glutamatergic neurons to control learning. *Cell Rep.* **38**, (2022).
92. Beier, K. T. *et al.* Circuit Architecture of VTA Dopamine Neurons Revealed by Systematic Input-Output Mapping. *Cell* **162**, 622–634 (2015).
93. Dewa, K.-I. *et al.* The astrocytic ensemble acts as a multiday trace to stabilize memory. *Nature* **648**, 146–156 (2025).

94. Zhang, X., Flick, K., Rizzo, M., Pignatelli, M. & Tonegawa, S. Dopamine induces fear extinction by activating the reward-responding amygdala neurons. *Proc. Natl. Acad. Sci. U. S. A.* **122**, e2501331122 (2025).
95. Tasaka, G.-I. *et al.* Orbitofrontal cortex influences dopamine dynamics associated with alloparental behavioral acquisition in female mice. *Sci. Adv.* **11**, eadr4620 (2025).
96. Dai, B. *et al.* Experience-dependent dopamine modulation of male aggression. *Nature* **639**, 430–437 (2025).
97. Xin, Q. *et al.* Neuron-astrocyte coupling in lateral habenula mediates depressive-like behaviors. *Cell* **188**, 3291-3309.e24 (2025).
98. Zhang, S. X. *et al.* Hypothalamic dopamine neurons motivate mating through persistent cAMP signalling. *Nature* **597**, 245–249 (2021).
99. Harada, M., Capdevila, L. S., Wilhelm, M., Burdakov, D. & Patriarchi, T. Stimulation of VTA dopamine inputs to LH upregulates orexin neuronal activity in a DRD2-dependent manner. *Elife* **12**, (2024).
100. Matosevich, N. *et al.* An early surge of norepinephrine along brainstem pathways drives sensory-evoked awakening. *Sci. Adv.* **11**, eadw6375 (2025).
101. Li, L. *et al.* Activity-dependent constraints on catecholamine signaling. *Cell Rep.* **42**, (2023).
102. Osorio-Forero, A. *et al.* Infraslow noradrenergic locus coeruleus activity fluctuations are gatekeepers of the NREM-REM sleep cycle. *Nat. Neurosci.* **28**, 84–96 (2025).
103. Agster, K. L., Mejias-Aponte, C. A., Clark, B. D. & Waterhouse, B. D. Evidence for a regional specificity in the density and distribution of noradrenergic varicosities in rat cortex. *J. Comp. Neurol.* **521**, 2195–2207 (2013).
104. Augustine, V. *et al.* Temporally and Spatially Distinct Thirst Satiation Signals. *Neuron* **103**, 242-249.e4 (2019).
105. Bayless, D. W. *et al.* A neural circuit for male sexual behavior and reward. *Cell* **186**, 3862-3881.e28 (2023).
106. de Jong, J. W. *et al.* A Neural Circuit Mechanism for Encoding Aversive Stimuli in the Mesolimbic Dopamine System. *Neuron* **101**, 133-151.e7 (2019).
107. Mohebi, A., Wei, W., Pelattini, L., Kim, K. & Berke, J. D. Dopamine transients follow a striatal gradient of reward time horizons. *Nat. Neurosci.* **27**, 737–746 (2024).
108. Patriarchi, T. *et al.* An expanded palette of dopamine sensors for multiplex imaging in vivo. *Nat. Methods* **17**, 1147–1155 (2020).
109. Salinas-Hernández, X. I., Zafiri, D., Sigurdsson, T. & Duvarci, S. Functional architecture of dopamine neurons driving fear extinction learning. *Neuron* **111**, 3854-3870.e5 (2023).
110. Vu, M. A. T. *et al.* Targeted micro-fiber arrays for measuring and manipulating localized multi-scale neural dynamics over large, deep brain volumes during behavior. *Neuron* **112**, 909-923.e9 (2024).
111. Liebana, S. *et al.* Dopamine encodes deep network teaching signals for individual learning trajectories. *Cell* **188**, 3789-3805.e33 (2025).
112. Liu, H. *et al.* Subsecond dopamine fluctuations do not specify the vigor of ongoing actions. *Nat. Neurosci.* **28**, 2432–2438 (2025).
113. Greenstreet, F. *et al.* Dopaminergic action prediction errors serve as a value-free teaching signal. *Nature* **643**, 1333–1342 (2025).
114. Molinari, M. *et al.* Dopamine and serotonin cotransmission filters striatonigral synaptic activity via 5-HT1B receptor activation. *Sci. Adv.* **11**, eadx4577 (2025).
115. Miyasaka, A. *et al.* Sequential transitions of male sexual behaviors driven by dual acetylcholine-dopamine dynamics. *Neuron* **113**, 1240-1258.e10 (2025).
116. Costa, K. M. *et al.* Dopamine and acetylcholine correlations in the nucleus accumbens depend on behavioral task states. *Curr. Biol.* **35**, 1400-1407.e3 (2025).

117. Gomez, J. L. *et al.* Cocaine chemogenetics blunts drug-seeking by synthetic physiology. *Nature* **646**, 746–753 (2025).
118. Sun, F. *et al.* Shared neural substrates of prosocial and parenting behaviours. *Nature* doi:10.1038/s41586-026-10327-8 (2026).
119. Onimus, O. *et al.* The gut-brain vagal axis governs mesolimbic dopamine dynamics and reward events. *Sci. Adv.* **12**, eadz0828 (2026).
120. Lebowitz, J. J., Banerjee, A., Handy, G., Williams, J. T. & Kaeser, P. S. The synaptic vesicle priming protein Munc13 mediates evoked somatodendritic dopamine release. *The Journal of Neuroscience* **in press**, (2026).
121. Beyene, A. G. *et al.* Imaging striatal dopamine release using a nongenetically encoded near infrared fluorescent catecholamine nanosensor. *Sci. Adv.* **5**, eaaw3108 (2019).
122. Qian, T. *et al.* A genetically encoded sensor measures temporal oxytocin release from different neuronal compartments. *Nat. Biotechnol.* **41**, 944–957 (2023).
123. Marvin, J. S. *et al.* An optimized fluorescent probe for visualizing glutamate neurotransmission. *Nat. Methods* **10**, 162–70 (2013).
124. Aggarwal, A. *et al.* Glutamate indicators with improved activation kinetics and localization for imaging synaptic transmission. *Nat. Methods* **20**, 925–934 (2023).

Supplementary table. Statistical tests and p-values for all figures

Figure	Panel	Statistical test	Comparison	p-value	F-values (if applicable)	Multiple comparisons (if applicable)		Confidence Intervals (if applicable)
						Comparison	p-value	
Fig. 1	c	Mann-Whitney rank-sum	M1 cortex vs Dorsal striatum	$p < 0.0001$				
	d	Mann-Whitney rank-sum	M1 cortex vs Dorsal striatum	$p < 0.0001$				
	h	Wilcoxon signed-rank	1 vs 20 stimuli	$p = 0.0002$				
	k	Wilcoxon signed-rank	1 vs 20 stimuli	$p = 0.3$				
	o	Wilcoxon signed-rank	1 vs 20 stimuli	$p = 0.002$				
	r	Paired t-test	1 vs 20 stimuli	$p = 0.6$				
Fig. 2	e (1 stimulus)	Mann-Whitney rank-sum	RIM control vs RIM cKO^{DA}	$p < 0.0001$				
	e (20 stimuli)	Mann-Whitney rank-sum	RIM control vs RIM cKO^{DA}	$p < 0.0001$				
	h (1 stimulus)	Mann-Whitney rank-sum	RIM control vs RIM cKO^{DA}	$p < 0.0001$				
	h (20 stimuli)	Mann-Whitney rank-sum	RIM control vs RIM cKO^{DA}	$p < 0.0001$				
	k ($GRAB_{NE}$)	Unpaired t-test	RIM control vs RIM cKO^{DA}	$p < 0.0001$				
Fig. 3	c	Mann-Whitney rank-sum	Control vs. 6-OHDA	$p < 0.0001$				
	g (1 stimulus)	Mann-Whitney rank-sum	Control vs. 6-OHDA	$p < 0.0001$				
	g (20 stimuli)	Mann-Whitney rank-sum	Control vs. 6-OHDA	$p < 0.0001$				
	k (1 stimulus)	Mann-Whitney rank-sum	Control vs. 6-OHDA	$p < 0.0001$				
	k (20 stimuli)	Mann-Whitney rank-sum	Control vs. 6-OHDA	$p < 0.0001$				
	o (1 stimulus)	Mann-Whitney rank-sum	Control vs. 6-OHDA	$p < 0.0001$				
	o (20 stimuli)	Mann-Whitney rank-sum	Control vs. 6-OHDA	$p < 0.0001$				
Extended data fig. 3	d (1 stimulus)	Wilcoxon signed-rank	Before TTX wash-on vs. After TTX wash-on	$p = 0.004$				
	d (20 stimuli)	Wilcoxon signed-rank	Before TTX wash-on vs. After TTX wash-on	$p = 0.004$				

	h (1 stimulus)	Wilcoxon signed-rank	Before cocktail wash-on vs. After cocktail wash-on	p = 0.03				
	h (20 stimuli)	Wilcoxon signed-rank	Before cocktail wash-on vs. After cocktail wash-on	p = 0.002				
	l (1 stimulus)	Paired t-test	Before vs. After	p = 0.04				
	l (20 stimuli)	Paired t-test	Before vs. After	p = 0.0002				
	m	One-way ANOVA	ACSF vs. TTX vs. Cocktail	$p < 0.0001$	F = 29.11, R^2 = 0.6913	ACSF vs. TTX	$p < 0.0001$	-88.85 to -43.38
						ACSF vs. Cocktail	p = 0.9	-24.94 to -19.33
	n	Kruskal-Wallis	ACSF vs. TTX vs. Cocktail	$p < 0.0001$		ACSF vs. TTX	$p < 0.0001$	
						ACSF vs. Cocktail	p = 0.5	
Extended data fig. 4	c	Mann-Whitney rank-sum	dopamine vs. norepinephrine	$p < 0.0001$				
	f	Mann-Whitney rank-sum	dopamine vs. norepinephrine	p = 0.7				
Extended data fig. 5	d (1 stimulus)	Mann-Whitney rank-sum	RIM control vs RIM cKO^{DA}	$p < 0.0001$				
	d (20 stimuli)	Mann-Whitney rank-sum	RIM control vs RIM cKO^{DA}	$p < 0.0001$				
Extended data fig. 7	f (1 stimulus)	Mann-Whitney rank-sum	Control vs. 6-OHDA	$p < 0.0001$				
	f (20 stimuli)	Mann-Whitney rank-sum	Control vs. 6-OHDA	$p < 0.0001$				
Extended data fig. 8	d (1 stimulus)	Unpaired t-test	Control vs. 6-OHDA	p = 0.8				
	d (20 stimuli)	Unpaired t-test	Control vs. 6-OHDA	p = 0.9				
Extended data fig. 9	d	Wilcoxon signed-rank	1 vs 20 stimuli	p = 0.03				
	g	Wilcoxon signed-rank	1 vs 20 stimuli	p = 0.03				
Extended data fig. 10	c	Mann-Whitney rank-sum	Control vs. 6-OHDA	$p < 0.0001$				
	d	Mann-Whitney rank-sum	Control vs. 6-OHDA	p = 0.9				
	h (1 stimulus)	Mann-Whitney rank-sum	Control vs. 6-OHDA	P = 0.002				
	h (20 stimuli)	Mann-Whitney rank-sum	Control vs. 6-OHDA	$p < 0.0001$				
	l (1 stimulus)	Mann-Whitney rank-sum	Control vs. 6-OHDA	p = 0.06				
	l (20 stimuli)	Mann-Whitney rank-sum	Control vs. 6-OHDA	p = 0.2				
