# Supplementary material for: Norepinephrine and dopamine sensor crosstalk depends on local innervation density
Source: Nat Neurosci. 2026 Aug 5;29(9):2103–9. doi: 10.1038/s41593-026-02379-w (PMC13533832; doi:10.1038/s41593-026-02379-w)
Supplement: Supplementary file 2 [file 41593_2026_2379_MOESM2_ESM.pdf]

nature portfolio

Corresponding author(s): Pascal S. Kaeser

Last updated by author(s): Jul 23, 2026

Reporting Summary

Nature Portfolio wishes to improve the reproducibility of the work that we publish. This form provides structure for consistency and transparency in reporting. For further information on Nature Portfolio policies, see our Editorial Policies and the Editorial Policy Checklist.

Statistics

For all statistical analyses, confirm that the following items are present in the figure legend, table legend, main text, or Methods section.

n/a	Confirmed	
☐	☒	The exact sample size (*n*) for each experimental group/condition, given as a discrete number and unit of measurement
☐	☒	A statement on whether measurements were taken from distinct samples or whether the same sample was measured repeatedly
☐	☒	The statistical test(s) used AND whether they are one- or two-sided<br>*Only common tests should be described solely by name; describe more complex techniques in the Methods section.*
☒	☐	A description of all covariates tested
☐	☒	A description of any assumptions or corrections, such as tests of normality and adjustment for multiple comparisons
☐	☒	A full description of the statistical parameters including central tendency (e.g. means) or other basic estimates (e.g. regression coefficient) AND variation (e.g. standard deviation) or associated estimates of uncertainty (e.g. confidence intervals)
☐	☒	For null hypothesis testing, the test statistic (e.g. *F*, *t*, *r*) with confidence intervals, effect sizes, degrees of freedom and *P* value noted<br>*Give P values as exact values whenever suitable.*
☒	☐	For Bayesian analysis, information on the choice of priors and Markov chain Monte Carlo settings
☒	☐	For hierarchical and complex designs, identification of the appropriate level for tests and full reporting of outcomes
☒	☐	Estimates of effect sizes (e.g. Cohen's *d*, Pearson's *r*), indicating how they were calculated

Our web collection on statistics for biologists contains articles on many of the points above.

Software and code

Policy information about availability of computer code

Data collection	Commercially available software was used for data acquisition (HCImage 4.2.6.1, ThorCam 3.6.0.6, MATLAB R2025b) as established previously (PMIDs: 39415006, 35324301) and as outlined in the methods section.
Data analysis	Commercially available software and open-source software (MATLAB R2025b, Python 3.11.4, GraphPad 10.6.1, ilastik 1.4.1.post1) were used for data analyses as established previously (PMIDs: 39415006, 35324301, 31570887) and as outlined in the methods section. For statistics, GraphPad Prism 10.6.1 was used as described in the methods section.

For manuscripts utilizing custom algorithms or software that are central to the research but not yet described in published literature, software must be made available to editors and reviewers. We strongly encourage code deposition in a community repository (e.g. GitHub). See the Nature Portfolio guidelines for submitting code & software for further information.

Data

Policy information about availability of data

All manuscripts must include a data availability statement. This statement should provide the following information, where applicable:

- Accession codes, unique identifiers, or web links for publicly available datasets
- A description of any restrictions on data availability
- For clinical datasets or third party data, please ensure that the statement adheres to our policy

Individual data points generated for this study are included in the figures whenever possible. Tabulated data are available at Zenodo, DOI: 10.5281/zenodo.20255734. Additional data are available from the corresponding author upon request.

Research involving human participants, their data, or biological material

Policy information about studies with human participants or human data. See also policy information about sex, gender (identity/presentation), and sexual orientation and race, ethnicity and racism.

Reporting on sex and gender	Not applicable
Reporting on race, ethnicity, or other socially relevant groupings	Not applicable
Population characteristics	Not applicable
Recruitment	Not applicable
Ethics oversight	Not applicable

Note that full information on the approval of the study protocol must also be provided in the manuscript.

Field-specific reporting

Please select the one below that is the best fit for your research. If you are not sure, read the appropriate sections before making your selection.

☒ Life sciences ☐ Behavioural & social sciences ☐ Ecological, evolutionary & environmental sciences

For a reference copy of the document with all sections, see nature.com/documents/nr-reporting-summary-flat.pdf

Life sciences study design

All studies must disclose on these points even when the disclosure is negative.

Sample size	Sample sizes were selected based on prior studies and are similar to existing, published studies in the field (PMIDs: 39415006, 35324301, 29398114, 32490813, 38547869, 38036855).
Data exclusions	Data that met quality standards as described in the methods section were included, and no outliers were excluded for genotype comparisons.
Replication	The number of observations is reported in each figure. For quantitative experiments using fixed brain slices, acute brain slices, or live animals, a minimum of three mice were used. For experiments in HEK293T cells, data were acquired from three independent transfections.
Randomization	Data were not randomized but pooled by genotype and/or condition after analyses by a blinded experimenter were completed.
Blinding	For experiments involving genotype comparisons, the experimenter was blind to genotype during data acquisition and analyses.

Reporting for specific materials, systems and methods

We require information from authors about some types of materials, experimental systems and methods used in many studies. Here, indicate whether each material, system or method listed is relevant to your study. If you are not sure if a list item applies to your research, read the appropriate section before selecting a response.

Materials & experimental systems

n/a	Involved in the study	
☐	☒	Antibodies
☐	☒	Eukaryotic cell lines
☒	☐	Palaeontology and archaeology
☐	☒	Animals and other organisms
☒	☐	Clinical data
☒	☐	Dual use research of concern
☒	☐	Plants

Methods

n/a	Involved in the study	
☒	☐	ChIP-seq
☒	☐	Flow cytometry
☒	☐	MRI-based neuroimaging

Antibodies

Antibodies used	The following primary antibodies were used: mouse IgG1 anti-NET (1:1000 Synaptic Systems #260 011, RRID:AB_2636907, lab antibody code A265), guinea pig anti-RFP (1:1000, Synaptic Systems #390 004, RRID:AB_2737052, A304), and rabbit anti-TH (1:2000, Millipore #AB152, RRID:AB_390204, A66). The following secondary antibodies were used: Alexa Fluor 405 goat anti-rabbit (1:500, Thermo Fisher Scientific #A-31556, RRID:AB_221605, S1), Alexa Fluor 488 goat anti-mouse IgG1 (1:500, Thermo Fisher Scientific #A-21121, RRID:AB_2535764, S7), and Alexa Fluor 568 goat anti guinea pig (1:500, Thermo Fisher Scientific #A-11075, RRID:AB_2534119, S27). Information for each antibody can be found on the manufacturer's website under the corresponding catalog numbers.
Validation	Antibodies were validated through the following observations: anti-NET signals overlap with fluorescent markers that are genetically expressed in the locus coeruleus. Both anti-NET and anti-TH signals are ablated upon chemical lesioning of the corresponding neurons with 6-OHDA. Anti-RFP signals are absent in brain areas or mice that do not express a protein tagged with a corresponding red fluorophore. Fluorophore-conjugated secondary antibodies display minimal background signal when applied in the absence of primary antibodies.

Eukaryotic cell lines

Policy information about cell lines and Sex and Gender in Research

Cell line source(s)	HEK293T cells were purchased from ATCC (Cat# CRL-3216; RRID: CVCL_0064).
Authentication	HEK293T cells were purchased from ATCC as a validated cell line. We did not further authenticate them in the laboratory.
Mycoplasma contamination	HEK293T cells were purchased from ATCC as a mycoplasma-free cell line.
Commonly misidentified lines (See ICLAC register)	None

Animals and other research organisms

Policy information about studies involving animals; ARRIVE guidelines recommended for reporting animal research, and Sex and Gender in Research

Laboratory animals	DATIRES-Cre mice (RRID:IMSR_JAX:006660, B6.SJL-Slc6a3tm1.1(cre)Bkmn/J, described in PMID: 16865686), Synaptophysin-tdTomato mice (RRID: IMSR_JAX:012570, B6;129S-Gt(ROSA)26Sortm34.1(CAG-Syp/tdTomato)Hze/J. described in PMID: 29398114), and conditional RIM1 (RRID:IMSR_JAX:015832, Rims1tm3Sud/J, described in PMID: 19074017) and RIM2 knockout mice (RRID:IMSR_JAX:015833, Rims2tm1.1Sud/J, described in PMID: 21241895) were used. Mice were maintained on a mixed background (containing C57BL/6 and 129/Sv) and housed in a room set to 20-24 °C and 50% humidity. Mice used for fiber photometry were housed in a reversed light-dark cycle, and behavioral experiments were performed during the dark phases.
Wild animals	The study does not include wild animals.
Reporting on sex	Female and male mice were included in all experiments irrespective of sex.
Field-collected samples	The study does not include samples collected from the field.
Ethics oversight	Animal experiments were performed in accordance with approved protocols of the Harvard University Animal Care and Use Committee.

Note that full information on the approval of the study protocol must also be provided in the manuscript.

Plants

Seed stocks	Not applicable.
Novel plant genotypes	Not applicable.
Authentication	Not applicable.
